# Candidate genes for infertility: an in-silico study based on cytogenetic analysis

**DOI:** 10.1186/s12920-022-01320-x

**Published:** 2022-08-02

**Authors:** Jatinder Singh Sahota, Bhavna Sharma, Kamlesh Guleria, Vasudha Sambyal

**Affiliations:** Department of Human Genetics, Cytogenetics Laboratory, Guru Nanak Dev University (GNDU), Amritsar, Punjab 143005 India

**Keywords:** Male infertility, Female infertility, Karyotyping, In-silico, Network analysis, Protein–protein interaction

## Abstract

**Background:**

The cause of infertility remains unclear in a significant proportion of reproductive-age couples who fail to conceive naturally. Chromosomal aberrations have been identified as one of the main genetic causes of male and female infertility. Structural chromosomal aberrations may disrupt the functioning of various genes, some of which may be important for fertility. The present study aims to identify candidate genes and putative functional interaction networks involved in male and female infertility using cytogenetic data from cultured peripheral blood lymphocytes of infertile patients.

**Methods:**

Karyotypic analyses was done in 201 infertile patients (100 males and 101 females) and 201 age and gender matched healthy controls (100 males and 101 females) after 72 h peripheral lymphocyte culturing and GTG banding, followed by bioinformatic analysis using Cytoscape v3.8.2 and Metascape.

**Results:**

Several chromosomal regions with a significantly higher frequency of structural aberrations were identified in the infertile males (5q2, 10q2, and 17q2) and females (6q2, 16q2, and Xq2). Segregation of the patients based on type of infertility (primary v/s secondary infertility) led to the identification of chromosomal regions with a significantly higher frequency of structural aberrations exclusively within the infertile males (5q2, 17q2) and females (16q2) with primary infertility. Cytoscape identified two networks specific to these regions: a male specific network with 99 genes and a female specific network with 109 genes. The top enriched GO terms within the male and female infertility networks were “skeletal system morphogenesis” and “mRNA transport” respectively. *PSME3*, *PSMD3*, and *CDC27* were the top 3 hub genes identified within the male infertility network. Similarly, *UPF3B*, *IRF8*, and *PSMB1* were the top 3 hub genes identified with the female infertility network. Among the hub genes identified in the male- and female-specific networks, *PSMB1*, *PSMD3*, and *PSME3 *are functional components of the proteasome complex. These hub genes have a limited number of reports related to their respective roles in maintenance of fertility in mice model and humans and require validation in further studies.

**Conclusion:**

The candidate genes predicted in the present study can serve as targets for future research on infertility.

**Supplementary Information:**

The online version contains supplementary material available at 10.1186/s12920-022-01320-x.

## Background

Human beings, despite a substantial growth in their population, can be considered a relatively infertile species [1]. Infertility is defined as a condition marked by the inability of a couple to conceive after one year of unprotected intercourse [2].

An estimated 8% to 12% couples of reproductive age worldwide are affected by infertility. Approximately 50% of total cases involve a diagnosis of infertility in the male partner [2, 3]. Male infertility can be caused by various mechanical, lifestyle and genetic factors. The genetic causes of male infertility include structural and numerical chromosome abnormalities, Y chromosome deletions, single gene disorders, and multifactorial causes [2]. Among the genetic factors, chromosomal anomalies have been identified as one of the main causes of male infertility [4]. With at least 2000 genes believed to be involved in spermatogenesis, the number of genetic anomalies associated with male infertility is growing steadily [5].

On the other hand, female infertility can be caused by developmental, endocrine, immunological, metabolic, microbial, surgical or genetic factors [6, 7]. The genetic causes of female infertility include chromosomal aberrations due to meiotic non-disjunction errors, copy number variants (CNV’s), single gene disorders and polygenic disorders [7].

Cytogenetic aberrations are included among the main genetic causes of infertility [4, 8]. Therefore, identification of chromosomal loci frequently involved in aberrations can help in identifying the genes/pathways involved in infertility using in-silico tools. Keeping these facts in mind, the present study uses a combination of cytogenetics and bioinformatic tools for the prediction of candidate genes which are actively involved in the pathogenesis of infertility.

## Methods

### Cytogenetic analysis

In order to study the cytogenetic aberrations associated with infertility, 201 infertile patients (100 males and 101 females) and 201 age and gender matched controls (100 males and 101 females) from a North-Indian population of Punjab, India were karyotyped after 72 h peripheral blood lymphocyte culturing and GTG-banding. The inclusion and exclusion criteria for recruitment of the infertile patients are given in Additional file 1: Table S1. The phenotypic presentations of the infertile males and females are given in Additional file 1: Table S2. The patients included in the present study were clinically diagnosed as infertile after failure to conceive via natural methods, with medications and also experiencing in-vitro fertilization (IVF) failure. These include a subset of patients wherein the fertility assessment parameters (spermiogram in males, hormonal profiles and reproductive imaging in females) were within the standard clinical limits in both the male and female partners undergoing IVF (Additional file 1: Table S2).

The cytogenetic analysis of the cultured peripheral blood lymphocytes of the patients and controls involved scoring of chromosomal aberrations as total metaphases showing any chromosomal aberration (TAM), metaphases showing only numerical aberrations (TMNA), metaphases showing only structural aberrations (TMSA), and metaphases showing both structural and numerical aberrations (TM(NA + SA)) in 50 to 100 metaphases per subject. The comparison of frequency of chromosomal aberrations in cases and controls was done using Student’s t-test. The cut off p-value adopted for statistical significance was 0.05.

### Bioinformatic analysis

The cytogenetic analysis helped in the identification of several chromosomal regions with a significantly higher frequency of structural aberrations among the infertile patients as compared to controls. The genes harbored within these loci were assessed by *in-silico* tools to predict candidate genes and pathways which might be impaired in infertile males and females. The cytogenetic loci observed within these regions were used as the input query for National Centre for Biotechnology Information (NCBI) Gene database (https://www.ncbi.nlm.nih.gov/gene) to identify the constituent genes. The data provided by NCBI Gene was filtered according to species (“*Homo sapiens*”), chromosome number (chromosomal regions not queried were removed) and number of exons (only genes containing one or more exons were included).

Cytoscape v3.8.2 [9] was used to generate various interactive biological networks from the genes annotated to the different chromosomal regions. In the present study, the ‘Gene Set/Mutation Analysis’ tool of the ‘Reactome Functional Interaction (FI)’ Cytoscape plugin [10] was used to generate the different interaction networks. For this purpose, the 2019 ‘Reactome FI Network’ dataset and ‘Show genes not linked to others’ options were used to create interaction networks without the addition of any linker gene. The cytoHubba plugin [11] within Cytoscape was used to identify the various hub genes within the male and female networks. The set of genes located within the different networks was used as the input for the web-based tool, Metascape [12], to identify the genes and pathways enriched within the infertile males and females.

## Results

### Cytogenetic analysis

The comparison of chromosomal aberrations between the infertile cases and age-matched controls revealed a significantly higher mean frequency of aberrations among the infertile cases (Table 1). A similar trend was observed upon segregating the cases and controls by gender and type of infertility (primary versus secondary infertility) (Tables 2, 3). Among the infertile patients, 5 males and 8 females were identified as carriers of constitutional anomalies. These patients were removed from further analysis resulting in 188 infertile patients (95 males and 93 females) and 188 age-matched controls (95 males and 93 females) remaining for further analysis. A significantly high mean frequency of structural aberrations (deletions, chromatid/chromosomal breaks and gaps) was identified in certain chromosomal regions within these subsets of patients (Table 4). The representative karyotypes for a subset of infertile patients and healthy controls are depicted in Additional file 1: Table S3.

**Table 1:** Cytogenetic profile of infertile cases and healthy controls

Variable	Male cases	Male controls	p-value	Female cases	Female controls	p-value
No. of subjects	100	100	–	101	101	–
Age (Mean ± SD) in years	34.61 ± 7.21	34.91 ± 7.66	0.7758	32.58 ± 6.28	34.88 ± 7.45	**0.0186**
Mean (%) aberrant metaphases	28.37 ± 13.46	11.28 ± 7.28	** < 0.0001**	29.16 ± 14.02	13.60 ± 7.03	** < 0.0001**
Mean (%) metaphases with structural aberrations	16.81 ± 10.99	5.44 ± 5.37	** < 0.0001**	17.94 ± 12.71	5.30 ± 4.37	** < 0.0001**
Mean (%) metaphases with numerical aberrations	7.82 ± 6.18	4.98 ± 3.98	**0.0002**	7.51 ± 5.32	7.03 ± 4.72	0.4984
Mean (%) metaphases with both structuraland numerical aberrations	3.57 ± 3.15	0.59 ± 1.01	** < 0.0001**	4.16 ± 3.80	1.43 ± 2.29	** < 0.0001**

Significant p-values (< 0.05), calculated by t-test, are shown in bold

**Table 2:** Comparison of cytogenetic profiles of primary infertility cases (male and female) with age and gender matched controls

Variable	Male primary infertility cases	Age-matched male controls	p-value	Female primary infertility cases	Age-matched female controls	p-value
No. of subjects	66	66	–	66	66	–
Age (Mean ± SD) in years	33.94 ± 6.93	34.24 ± 7.23	0.8081	31.80 ± 6.0	33.94 ± 7.28	0.0676
Mean (%) aberrant metaphases	26.96 ± 12.52	11.31 ± 7.02	** < 0.0001**	27.22 ± 12.34	13.44 ± 7.12	** < 0.0001**
Mean (%) metaphases with structural aberrations	16.17 ± 10.74	5.29 ± 5.06	** < 0.0001**	16.74 ± 11.87	5.42 ± 4.83	** < 0.0001**
Mean (%) metaphases with numerical aberrations	7.24 ± 5.50	5.34 ± 4.27	**0.0284**	7.4 ± 5.33	6.96 ± 4.80	0.6191
Mean (%) metaphases with both structural and numerical aberrations	3.25 ± 2.99	0.55 ± 1.04	** < 0.0001**	3.49 ± 2.9	1.34 ± 2.15	** < 0.0001**

Significant p-values (< 0.05), calculated by t-test, are shown in bold

**Table 3:** Comparison of cytogenetic profiles of secondary infertility cases (male and female) with age and gender-matched controls

Variable	Male secondary infertility cases	Age-matched male controls	p-value	Female secondary infertility cases	Age-matched female controls	p-value
No. of subjects	34	34	–	35	35	–
Age (Mean ± SD) in years	35.91 ± 7.66	36.21 ± 8.39	0.8781	34.06 ± 6.62	36.66 ± 7.55	0.1302
Mean (%) aberrant metaphases	31.12 ± 14.9	11.23 ± 7.88	** < 0.0001**	32.67 ± 16.24	13.91 ± 6.92	** < 0.0001**
Mean (%) metaphases with structural aberrations	18.29 ± 11.3	5.73 ± 5.99	** < 0.0001**	20.12 ± 14.01	5.06 ± 3.38	** < 0.0001**
Mean (%) metaphases with numerical aberrations	8.6 ± 7.4	4.29 ± 3.29	**0.0028**	7.51 ± 5.39	7.16 ± 4.62	0.7714
Mean (%) metaphases with both structural and numerical aberrations	4.3 ± 3.4	0.68 ± 0.94	** < 0.0001**	5.38 ± 4.85	1.60 ± 2.55	**0.0001**

Significant p-values (< 0.05), calculated by t-test, are shown in bold

**Table 4:** List of chromosomal regions with a significantly higher frequency of structural aberrations in infertile males and females

Gender	Chromosome/chromosomal arm/chromosomal region	Frequency of aberrations in infertility cases (Mean ± SD)	Frequency of aberrations in healthy age-matched controls (Mean ± SD)*	p-value
Male	5q2	1.67 ± 0.58	1.00 ± 0.00	** < 0.0001**
	10q2	1.50 ± 0.71	1.00 ± 0.00	** < 0.0001**
	17q2	1.33 ± 0.58	1.00 ± 0.00	** < 0.0001**
Female	6q2	1.17 ± 0.41	1.00 ± 0.00	** < 0.0001**
	16q2	1.50 ± 0.71	1.00 ± 0.00	** < 0.0001**
	Xq2	2.00 ± 1.18	1.00 ± 0.00	** < 0.0001**

Significant *p*-values (<0.05), calculated by t-test, are highlighted in bold

*The zero values were omitted during the calculation of Mean and Standard Deviation due to presence of a high number of zeros in the data

Upon segregating the patients based on type of infertility (primary vs. secondary infertility), chromosomal regions with a significantly high mean frequency of structural aberrations were identified only within the primary infertility patients (Table 5). The cytogenetic loci affected within these regions (Tables 4 and 5) were subjected to bioinformatic analysis.

**Table 5:** List of chromosomal regions with a significantly higher frequency of structural aberrations in males and females diagnosed with primary infertility

Gender	Chromosome/chromosomal arm/chromosomal region	Frequency of aberrations in infertility cases (Mean ± SD)	Frequency of aberrations in healthy age-matched controls (Mean ± SD)*	p-value
Male	5q2	1.50 ± 0.71	1.00 ± 0.00	** < 0.0001**
	17q2	1.50 ± 0.71	1.00 ± 0.00	** < 0.0001**
Female	16q2	1.50 ± 0.71	1.00 ± 0.00	** < 0.0001**

Significant *p*-values (<0.05), calculated by t-test, are highlighted in bold

*The zero values were omitted during the calculation of Mean and Standard Deviation due to presence of a high number of zeros in the data.

### Bioinformatic analysis

**Fig. 1:**
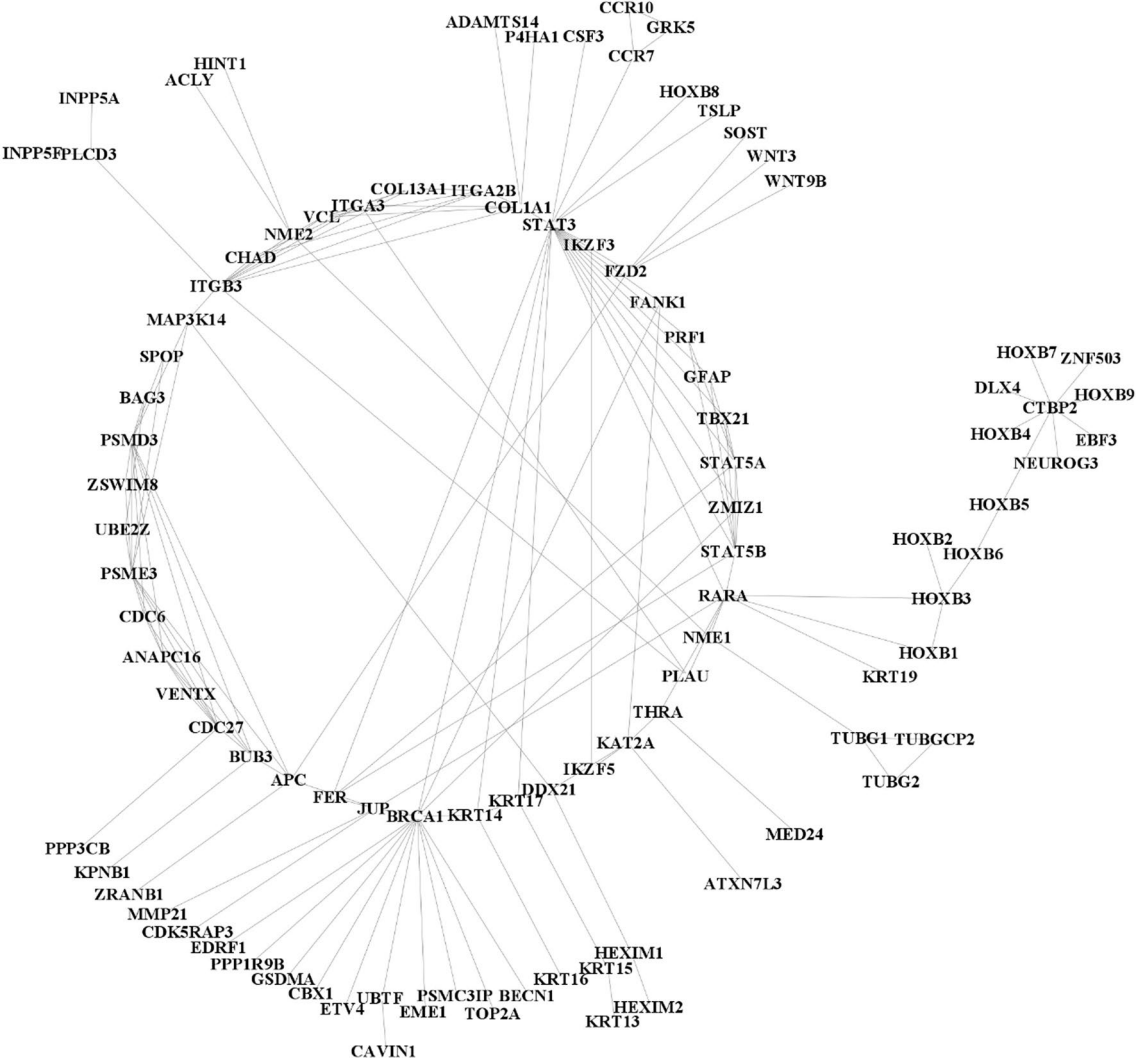
Biological interaction network generated using Cytoscape v3.8.2 for the male infertility dataset

**Fig. 2:**
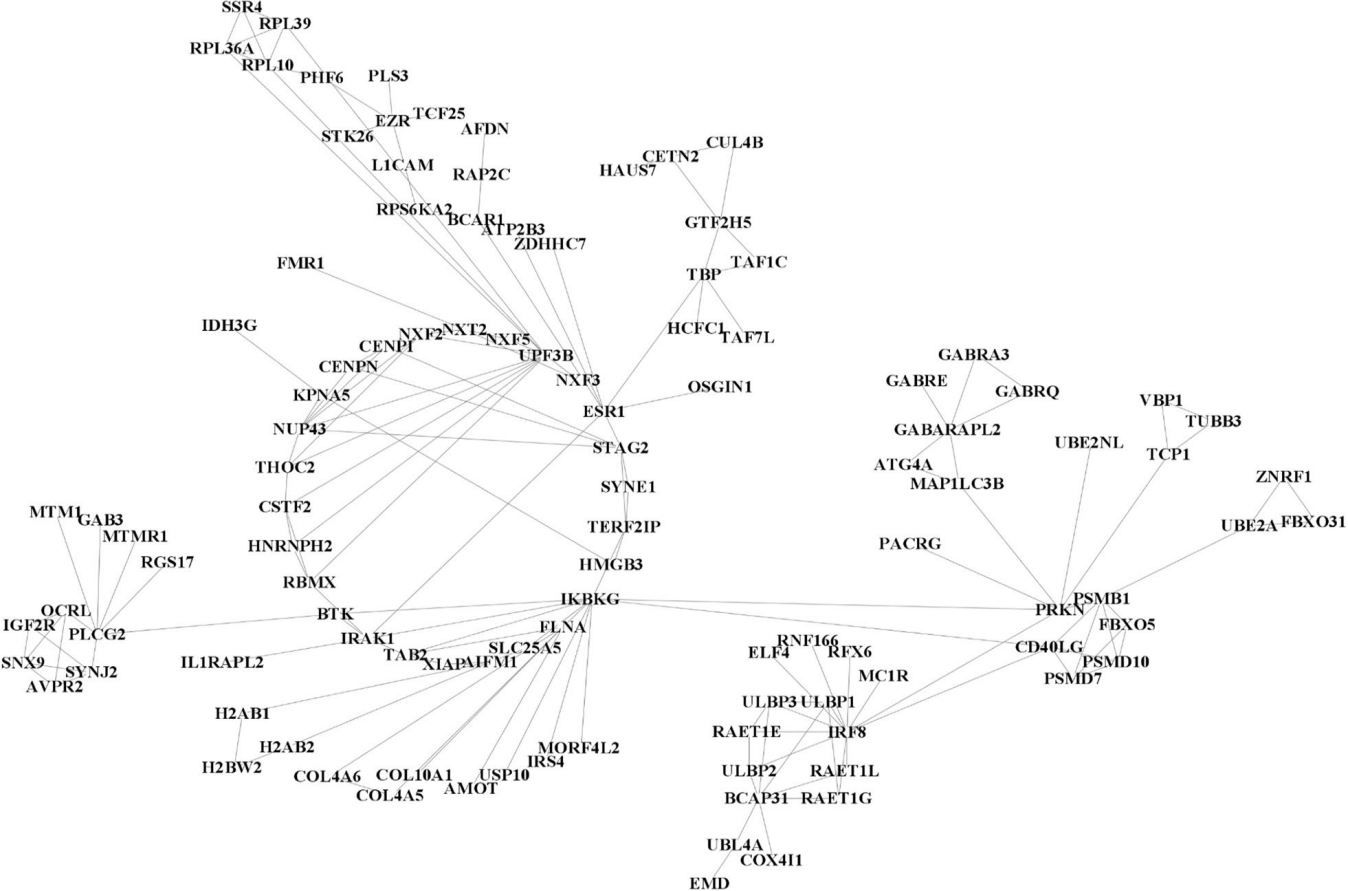
Biological interaction network generated using Cytoscape v3.8.2 for the female infertility dataset

**Fig. 3:**
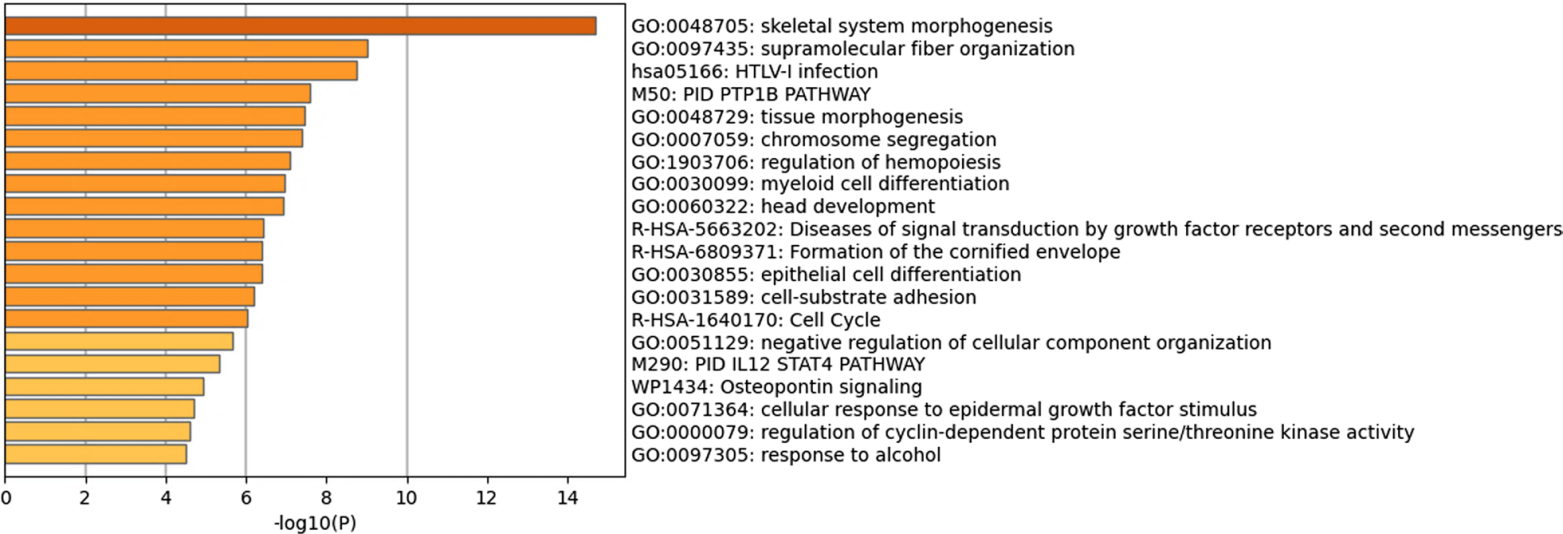
A list of the top 20 enriched gene ontology categories identified by Metascape for the male infertility dataset

**Fig. 4:**
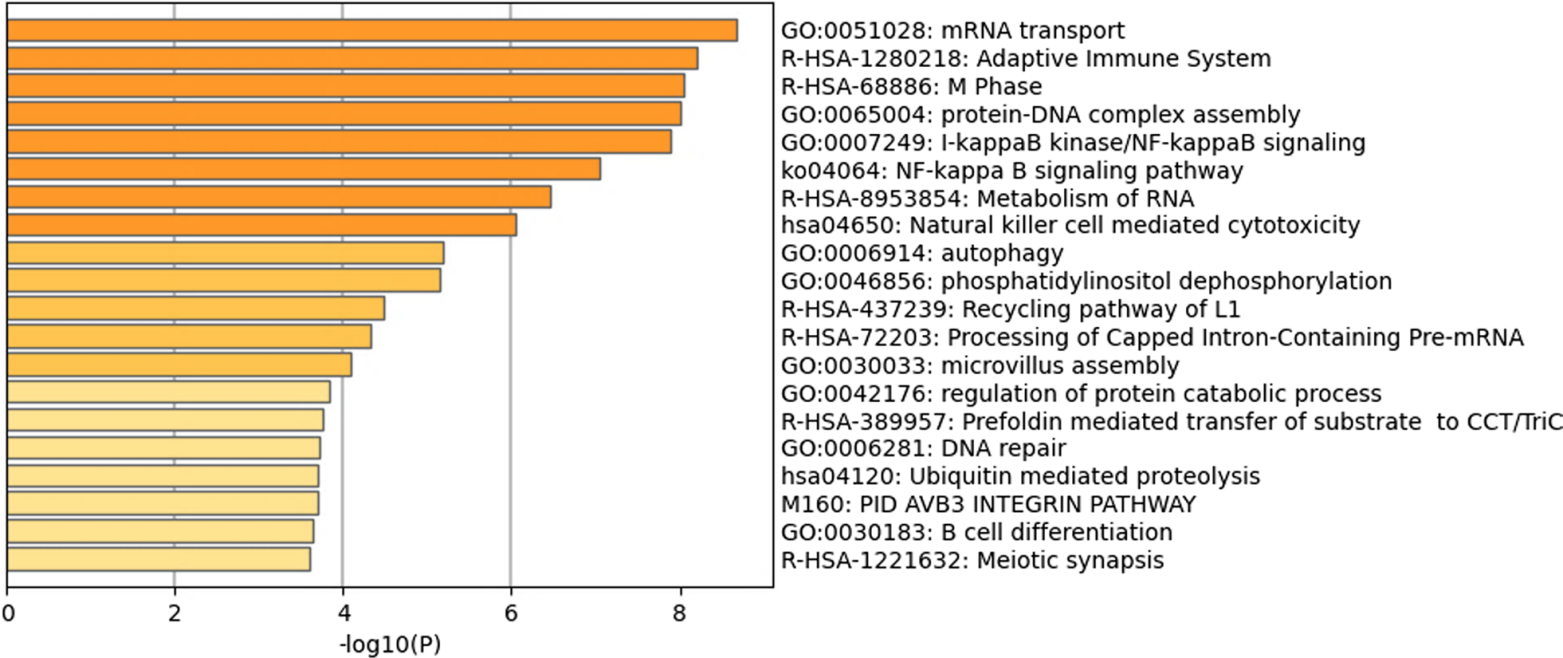
A list of the top 20 enriched gene ontology categories identified by Metascape for the female infertility dataset

NCBI Gene returned a list of the genes present at the cytogenetic loci queried: 731 genes in the male dataset and 901 genes in the female dataset. Querying Reactome FI with the aforementioned gene sets led to the generation of a network of 99 genes in the male-specific network (Fig. 1) and 109 genes in the female-specific network (Fig. 2). Further analysis by cytoHubba led to the identification of hub genes within the male (*PSMD3*, *PSME3*, and *CDC27*) and female (*UPF3B*, *IRF8*, and *PSMB1*) networks. Metascape identified “skeletal system morphogenesis” as the top enriched term within the male infertility network (Fig. 3) and “mRNA transport” as the top enriched term within the female infertility network (Fig. 4).

## Discussion

The human interactome is a highly complex network of functionally interacting cellular components, including a multitude of genes, proteins, metabolites, and RNA molecules [13]. It is now believed that many diseases manifest as a result of disruption of biological cascades due to altered interaction of various network components [14].

Among the structural aberrations identified in the present study, deletions, chromatid/chromosomal gaps and breaks were the most frequentin the infertile patients. In the present study, terminal deletions were observed in the 6q, 16q, and Xq region in infertile females. Deletions, either terminal or interstitial, result in loss of chromosomal segments and a subsequent haploinsufficiency of the gene(s) located in the deleted segments [15].Besides deletions, chromatid/chromosomal gaps and breaks were observed in both males (5q2, 10q2, and 17q2) and females (6q2 and Xq2). These aberrations occur as a consequence of DNA damage through exposure to physical and/or chemical agents, or as a result of recombination events [16]. If left unrepaired, chromosomal breaks can result in deletions (small- or large-scale) and translocations [17].

In the bio-informatic analysis in the present study, the top enriched gene ontology (GO) category within the male infertility network was GO:0048705—“skeletal system morphogenesis” (Fig. 3). The genes enriched within this category included *APC*, *BRCA1*, *CHAD*, *COL1A1*, *COL13A1*, *FZD2*, *HOXB1*, *HOXB2*, *HOXB3*, *HOXB4*, *HOXB5*, *HOXB6*, *HOXB7*, *HOXB8*, *HOXB9*, *ITGA3*, *ITGB3*, *KAT2A*, *KRT19*, *MMP21*, *NEUROG3*, *PLCD3*, *RARA*, *TBX21*, *THRA*, *WNT3*, *WNT9B*, *ZMIZ1*. Twenty-two genes enriched within this category have published reports on roles in maintenance of male fertility (*APC*, *BRCA1*, *COL1A1*, *COL13A1, FZD2, HOXB1, HOXB2, HOXB4, HOXB5, HOXB6, HOXB7, HOXB8, HOXB9, ITGA3, KAT2A*, *KRT19, NEUROG3, RARA, THRA, WNT3, WNT9B, ZMIZ1*) (Additional file 1: Table S4). Among the male infertility network, sixty-two genes had literature published on roles in male fertility (Additional file 1: Table S4).

The top enriched category within the female infertility network was GO:0051028—“mRNA transport” (Fig. 4). The genes enriched within this category included *CETN2*, *CSTF2*, *EMD*, *EZR, FLNA*, *FMR1*, *HCFC1*, *IKBKG*, *KPNA5*, *NUP43*, *NXF2*, *NXF3*, *NXF5*, *NXT2*, *SLC25A5, TAB2, TBP, TCP1, THOC2, UPF3B*. A dozen genes enriched within this category have published reports on roles in maintenance of female fertility (*CETN2, CSTF2, EZR, FLNA, FMR1, HCFC1, IKBKG, NUP43, NXF5, SLC25A5, TAB2, UPF3B*) (Additional file 1: Table S5). Among the female infertility network, sixty-eight genes had literature published on roles in female infertility (Additional file 1: Table S5).

In the male infertility network, the top 3 hub genes identified were *PSME3*, *PSMD3*, and *CDC27*. Research on mice models have shown that double knockout of *Psme3* and *Psme4* results in complete infertility in males [18]. In an additional report, male mice with *PSME3* (also known as *REGγ*) deficiency exhibited subfertility due to a decrease in the activity and concentration of spermatozoa [19]. The comparison of gene expression profiles of high motility sperm samples between healthy normozoospermic and asthenozoospermic individuals showed that *PSMD3*, *CDC27* and many other proteins involved in protein polyubiquitination were significantly downregulated in asthenozoospermic individuals [20]. The ubiquitin–proteasome system (UPS) has been reported to play an important role in sperm capacitation and fertilization [21]. Therefore, the UPS components involved in the sperm proteasome can be considered as potential candidates for further research on male infertility.

In the female infertility network, the top 3 hub genes identified were *UPF3B*, *IRF8* and *PSMB1*. Copy number variation in the 6q27 region (which includes *PSMB1*) have been speculated to be the cause of premature ovarian failure (POF) in a patient from a POF cohort [22]. In recent publications, *IRF8* positive cells were reported to be increased during the proliferative phase of the menstrual cycle in the endometrium of women with endometriosis [23]. Additionally, *IRF8* and *MEF2C* have been reported to be regulated at both mRNA and protein level in the endometrial epithelium during the window of implantation [24]. *Upf3b* was predicted to be a target gene for the rno-miR-141-5p microRNA. This miRNA was reported to possibly play a role in modulating endometrial receptivity in rats with endometriosis [25]. Currently, limited reports are available on the roles of these genes in maintenance of female fertility, warranting further research on these candidates.

Analysis of the predicted loss-of-function (pLOF) variants in the Genome Aggregation Database (gnomAD) browser [26] suggests that the hub genes, *CDC27*, *PSMD3*, *PSME3* (male-specific network), *PSMB1*, *UPF3B* (female-specific network) are intolerant to loss-of-function variants. In the clinical setting, microarray-based comparative genomic hybridization (aCGH) coupled with multiplex ligation-dependent probe amplification (MLPA) would be a better alternative to identify genomic imbalances within infertile patients having structural aberrations (especially deletions) within the chromosomal regions harboring these genes [27].

There are few limitations associated with the present study. A cytogenetic approach has been used in the present study to identify possible candidate genes located in chromosomal regions with a high mean frequency of structural aberrations in infertile patients, compared to healthy control individuals. GTG banding has been used for cytogenetic analysis. Compared to other microscopy-based alternatives, G-banding has a lower resolution [28].Finally, there is no expression-based data for the present dataset which can reveal the differentially expressed genes associated with the infertility subsets.

## Conclusion

The present study has identified several candidate genes associated with male and female infertility based on information of aberrations available from chromosomal analysis in G-banded cultured peripheral blood lymphocytes. Among the hub genes, the *PSMB1* (female-specific network), *PSMD3*, and *PSME3* (male-specifc network) are components of the proteasome complex. Currently, limited research has been conducted in human infertility on the roles of most of the genes predicted in the present study with a majority of the available reports limited to murine models. Therefore, future research may focus on determining the role of these genes in the maintenance of human fertility.

## Supplementary Information

**Additional file 1:** Clinical characteristics and karyotypes of study participants with supporting literature.

## Data Availability

All data generated or analyzed during this study are included in this article.

## Abbreviations

aCGH: Array-based comparative genomic hybridization
*CHAD*: Chondroadherin
CNV’s: Copy number variations
*EMD*: Emerin
*EZR*: Ezrin
FI: Functional interaction
gnomAD: Genome aggregation database
GO: Gene ontology
GTG: G-bands after trypsin and Giemsa
*HOXB3*: Homeobox B3
IVF: In-vitro fertilization
*KPNA5*: Karyopherin subunit alpha 5
*MEF2C*: Myocyte enhancer factor 2C
MLPA: Multiplex ligation-dependent probe amplification
*MMP21*: Matrix metallopeptidase 21
NCBI: National Centre for Biotechnology Information
*NXF2*: Nuclear RNA export factor 2
*NXF3*: Nuclear RNA export factor 3
*NXT2*: Nuclear transport factor 2 like export factor 2
*PALB2*: Partner and localizer of BRCA2
*PLCD3*: Phospholipase C delta 3
pLOF: Predicted loss-of function
POF: Premature ovarian failure
*STAT3*: Signal transducer and activator of transcription 3
T1LCs: T1 prospermatogonia-like cells
*TBP*: TATA-box binding protein
TAM: Total metaphases showing any chromosomal aberration
*TCP1*: T-complex 1
*THOC2*: THO complex 2
TM(NA + SA): Total metaphases showing both structural and numerical aberrations.
TMNA: Total metaphases showing only numerical aberrations
TMSA: Total metaphases showing only structural aberrations
UPS: Ubiquitin–proteasome system
