# Supplementary material for: Candidate genes for infertility: an in-silico study based on cytogenetic analysis
Source: BMC Med Genomics. 2022 Aug 2;15:170. doi: 10.1186/s12920-022-01320-x (PMC9347124; doi:10.1186/s12920-022-01320-x)
Supplement: Supplementary file 1 — Additional file 1: Clinical characteristics and karyotypes of study participants with supporting literature. [file 12920_2022_1320_MOESM1_ESM.docx]

**Supplementary Table 1: The inclusion and exclusion criteria for recruitment of patients and controls in the present study.**

| **Category** | **Patients** | **Controls** |
| --- | --- | --- |
| Inclusion criteria | Clinically confirmed infertile patients (Primary/Secondary Infertility) having no offspring. | -Age and gender matched healthy individuals having children conceived by natural methods residing in the same geographical area as that of the patients.  -No sign of any malignancy or chronic disease. |
| Exclusion criteria | Patients having single/multiple live offspring. | -Individuals suffering from any chronic or malignant disease.  -Individuals on regular medication.  -Individuals having conceived children using IVF/IUI/any other ART. |

**ART: Assisted Reproductive Technology; IUI: Intrauterine insemination; IVF: In Vitro Fertilization**

**Supplementary Table 2: Classification of the infertile patients based on clinical assessment.**

| **Infertile patients** | **Diagnosis** | **Number (%)** | **Additional conditions identified***  **Number (%)** |
| --- | --- | --- | --- |
| Infertile males | Males with normal sperm count | 78 (78.0) | Erectile dysfunction  1 (1.0)  Issues with ejaculation  2 (2.0) |
|  | Oligospermic males | 18 (18.0) | Issues with ejaculation  1 (1.0) |
|  | Azoospermic males | 4 (4.0) | - |
| Infertile females | Females with no apparent reproductive issue | 46 (45.54) | - |
|  | Polycystic ovary disease | 9 (8.91) | - |
|  | Uterine cysts/fibroids | 11 (10.89) | Menstrual irregularities  3 (2.97)  Tuberculosis infection  2 (1.98)  Blocked fallopian tubes  1 (0.99) |
|  | Tuberculosis infection | 5 (4.95) | Menstrual irregularities  1 (0.99) |
|  | Menstrual irregularities | 16 (15.84) | - |
|  | Blocked fallopian tubes | 7 (6.93) | Tuberculosis infection  3 (2.97) |
|  | Poor egg quality | 2 (1.98) | - |
|  | Endometriosis | 3 (2.97) | Tuberculosis infection  1 (0.99) |
|  | Ovarian cysts | 1 (0.99) | - |
|  | Abnormal uterine morphology | 1 (0.99) | - |

***A subset of infertile males and females had multiple reproductive issues and therefore could not be categorized into one single defined category.**

**Supplementary Table 3(a): Representative karyotypes for males with primary infertility harbouring the structural aberrations with increased frequency in infertile patients**

| **Males with Primary Infertility** | **TMS**  **Normal (%)**  **TAM (%)** | **Representative Karyotypes** | | |
| --- | --- | --- | --- | --- |
|  |  | **TMSA (%)** | **TMNA (%)** | **TM(SA+NA) (%)** |
| INF27B | 100  67 (67.0)  33 (33.0) | 23 (23.0)  46,XY,add(9)(q12q13)[5]  46,XY,chtb(15)(q24.2)  46,XY,chtg(6)(q26)  46,XY,chtg(12)(q13.2)  46,XY,chtg(17)(q21.3)  46,XY,chrb(5)(q23.3)  46,XY,chrb(13)(q14.2)  46,XY,chrb(13)(q14.2),del(Y)(p11.2)  45,XY,t(15;22)(15qter→15q10::22q10→22qter),tas(14;15)(14qter→14pter→15pter→15qter)  46,XY,tas(2;14)(2pter→2qter→14pter→14qter)  46,XY,tas(5;12)(5qter→5pter→12pter→12qter)  46,XY,tas(8;14)(8qter→8pter→14pter→14qter)  46,XY,tas(13;13)(13qter→13pter→13pter→13qter)  46,XY,tas(13;16)(13qter→13pter→16qter→16pter)  46,XY,tas(13;19)(13qter→13pter→19pter→19qter)  46,XY,tas(14;22)(14qter→14pter→22pter→22qter)  46,XY,tas(16;21)(16qter→16pter→21pter→21qter)  46,XY,tas(21;22)(21qter→21pter→22pter→22qter)  46,XY,tas(21;22)(21qter→21pter→22qter→22pter) | 7 (7.0)  46,XY,-1,+4  45,XY,-3,+4,-8  45,XY,-9  45,XY,-11  45,XY,-14  44,XY,-16,-17  45,XY,-22 | 3 (3.0)  47,XY,+4,-6,fra(19)(q13.2),+21  45,XY,+4,-8,-9,chrb(9)(9pter→cen→q21::frag::  q22→qter)  44,XY,-15,-22, +mar,+ace,+ace,+ace |
| INF39B | 70  44 (63.0)  26 (37.0) | 17 (24.28)  46,XY,add(9)(q12q13)[4]  46,XY,add(9)(q12q13),tas(13;14;21)(13qter→13pter→14pter→21pter→21qter)  46,XY,chtb(5)(q23.3),chtg(9)(q12)  46,XY,chtb(4)(q34.3)  46,XY,del(9)(pter→q31:)  45,XY,dic(14;21)(14qter→14p10::21p10→21qter)  45,XY,rob(13;15)(13qter→13q10::15q10→15qter)  46,XY,tas(8;19)(8qter→8pter→19pter→19qter),tas(15;9;13)(15qter→15pter→9pter→13pter→13qter),tas(13;22)(13qter→13pter→22pter→22qter)  46,XY,tas(8;21)(8qter→8pter→21pter→21qter)  46,XY,tas(11;21)(11pter→11qter→21pter→21qter)  46,XX,tas(13;14)(13qter→13pter→14pter→14qter),tas(18;20)(18qter→18pter→20pter→20qter)  46,XY,tas(13;14)(13qter→13pter→14pter→14qter)  46,XY,tas(13;21)(13qter→13pter→21pter→21qter)  46,XY,tas(13;21)(13qter→13pter→21pter→21qter),add(9)(q12q13) | 6 (8.57)  47,XYY,-19,+21  45,XY,-4  45,XY,-8  45,XY,-8,add(9)(q12)  44,Y,-X,-4  42,XY,-5,-7,-11,-13 | 3 (4.28)  45,XY,-8,add(9)(q12q13)  45,XY,tas(13;21)(13qter→13pter→21pter→21qter),-21  45,XY,tas(Y;21)(Yqter→Ypter→21qter→21pter),-9 |
| INF42B | 50  43 (86.0)  7 (14.0) | 5 (10.0)  46,XY,chtg(17)(q21)  45,XY,dic(13;14)(13qter→13q10::14q10→14qter),chtg(15)(q22)/46,Y,dic(X),tas(13;21)(13qter→13pter→21pter→21qter)  46,XY,tas(13;21)(13qter→13pter→21qter→21pter)  46,XY,tas(15;15)(15qter→15pter→15pter→15qter) | 2 (4.0)  43,XX,-9,-11,-22  45,XX,-18 | 0 (0.0) |
| INF56B | 50  43 (86.0)  7 (14.0) | 4 (8.0)  46,XY,chtb(4)(q21)  46,XY,chtb(5)(q21)  46,XY,tas(13;22)(13qter→13qter→22qter→22qter),tas(15;22)(15qter→15qter→22qter→22qter)  46,XX,tas(14;14)(14qter→14pter→14pter→14qter) | 3 (6.0)  45,XY,-13[2]  45,XY,-18 | 0 (0.0) |
| INF64B | 50  35 (70.0)  15 (30.0) | 7 (14.0)  46,XY,chtb(10)(q22)  46,XY,tas(13;22)(13qter→13pter→22pter→22qter)  46,XY,tas(14;21)(14qter→14pter→21pter→22qter)  46,XY,tas(15;21)(15qter→15pter→21pter→21qter)  46,XY,tas(20;22)(20qter→20pter→22pter→22qter)  46,XY,tas(21;22)(21qter→21pter→22pter→22qter)[2] | 4 (8.0)  46, XY,-2,+10  42,XY,-3,-11,-15,-21  45,XY,-18  38,Y,-X,-4,-11,-15,-16,-18,-21,-22 | 4 (8.0)  44,XX,-4,tas(6;18)(6qter→6pter →  18pter→18qter),-13  45,XY,-13,add(9)(q12q13)  44,XY,-16,-19,tas(21;22)(21qter →21pter→ 22pter→22qter)  45,XY,del(9)(q22),-11 |
| INF66B | 50  32 (64.0)  18 (36.0) | 13 (26.0)  46,XY,chtg(17)(q21)  45,XX,rob(15;21)(15qter→15p10::21p10→21qter)  46,XX,t(1;18)(1pter→1q12::18p11.8→18qter)  46,XY,tas(13;14)(13qter→13pter→14pter→14qter)  46,XY,tas(13;15)(13qter→13pter→15pter→15qter)[2]  46,XY,tas(13;21)(13qter→13pter→21pter→21qter),tas(13;14)(13qter→13pter→14pter→14qter)  46,XX,tas(13;22)(13qter→13pter→22pter→22qter)[2]  46,XY,tas(14;21)(14qter→14pter→21pter→21qter)  46,XY,tas(16;15)(16qter→16pter→15pter→15qter)  46,XX,tas(14;15)(14qter→14pter→15pter→15qter)  46,XY,ttr(13;14;14;21)(13qter→13p11→14p11→14p11→21p11) | 4 (8.0)  45,XY,-9  44,XY,-9,-20  44,XY,-10,-15  44,XY,-13,-17 | 1 (2.0)  45,XY,-11,tas(17;21)(17qter→17pter →21pter→21qter) |
| INF76B | 50  23 (46.0)  27 (54.0) | 23 (46.0)  46,XY,+ace  46,XY,chrb(1)(q21.2)  46,XY,chrb(1)(q21.2),tas(3;20)(3qter→3pter→20pter→20qter),tas(4;3)(4qter→4pter→3qter→3pter)  46,XY,chrb(1)(q21.2),tas(12;21)(12pter→12qter→21qter→21pter)  46,XY,chtb(2)(q14.3)  46,XY,tas(14;Y;13)(14qter→14pter→Ypter→13pter→13qter)  46,XY,chtg(8)(q21)  45,XY,chtb(8)(q21.3),rob(14;21)(14qter→14q10::21q10→21qter)  46,XY,chtb(10)(q22),+ace  46,XY,chtb(11)(p12),chtg(11)(q14.3)  46,XY,tas(4;8)(4pter→4qter→8qter→8pter)  46,XY,tas(8;9)(8qter→8pter→9pter→9qter),tas(13;22)(13qter→13pter→22pter→22qter)  46,XY,tas(10;15)(10qter→10pter→15pter→15qter),cht(b)(10)(p12.1)  46,XY,tas(13;22)(13qter→13pter→22pter→22qter),tas(8;10)(10qter→10pter→8qter→8pter)  46,XY,tas(14;15;21)(15qter→15pter→14pter→21pter→21qter)  46,XY,tas(14;21)(14qter→14pter→21pter→21qter)  46,XY,tas(15;22)(15qter→15pter→22pter→22qter)[3]  46,XY,tas(15;22;21)(15qter→15pter→22pter→21pter→21qter),tas(21;22)(21qter→21pter→22pter→22qter)  46,XY,tas(18;21)(18pter→18qter→21qter→21pter)  46,XY,tas(21;22)(21qter→21pter→22pter→22qter)  46,XY,tas(21;22)(21qter→21pter→22pter→22qter),tas(5;17)(5pter→5qter→17pter→17qter) | 3 (6.0)  41,XY,-7,-12,-19,-21,-22  45,XY,-8  45,XY,-22 | 1 (2.0)  45,XY,chrb(3)(p22),-14 |

**Supplementary Table 3(b): Representative karyotypes for females with primary infertility harbouring the structural aberrations with increased frequency in infertile patients**

| **Females with Primary Infertility** | **TMS**  **Normal (%)**  **TAM (%)** | **Representative Karyotypes** | | |
| --- | --- | --- | --- | --- |
|  |  | **TMSA (%)** | **TMNA (%)** | **TM(SA+NA) (%)** |
| INF12A | 50  40 (80.0)  10 (20.0) | 5 (10.0)  46,X,del(X)(q25)  46,XX,tas(3;21)(3qter→3pter→21pter→21qter),tas(13;22)(13qter→13pter→22pter→22qter)  46,XX,tas(13;22)(13qter→13pter→22pter→22qter)[2]  45,XX,t(21;22)(21qter→21q10::22q10→22qter) | 3 (6.0)  44, XX,-2,-10  44, XX,-9,-12  45,XX,-20 | 2 (4.0)  44,XX,-8,-12,tas(13;21)(13qter→13pter→ 21pter→21qter)  44,XX,tas(14;15)(14qter→14pter→15pter→15qter),dic(22;22)(22qter→22q10::22q10→22pter) |
| INF25A | 100  69 (69.0)  31 (31.0) | 27 (27.0)  46,XX,+ace,+ace  46,XX,add(9)(q12q13)[15]  46,XX,chtg(1)(q21)  46,XY,chtg(5)(q33.3)[3]  46,XX,del(13)(p11.2)  46,XX,dic(2;22)(2qter→2p36.3::22p13→22qter),13ps+ve  46,XX,fra(6)(q27)  46,XX,22ps+ve  46,XX,tas(2;21)(2qter→2pter→21pter→21qter),chrb(5)(q13)  46,XX,tas(13;14)(13qter→13pter→14pter→14qter)  46,XX,tas(15;15)(15qter→15pter→15pter→15qter),tas(3;19)(3pter→3qter→19pter→19qter) | 1 (1.0)  46,XX,+6,-11 | 3 (3.0)  44,XX,-1,add(9)(q12q13),-16  44,XX,add(9))q12q13),-16,-22  46,XX,inv(10) (q11q26.2),+21,-22 |
| INF31A | 60  32 (53.33)  28 (46.66) | 12 (20.0)  46,X,chtb(X)(q12)  46,XX,chtb(2)(q36)  46,XX,chtg(5)(q12)  46,XX,del(16)(pter→q23:)  end 46,XX  46,XX,inv(9)(q13q11)  pcd46,XX  45,XX,rob(13;14)(13qter→13q10::14q10→14qter)  45,XX,rob(14;14)(14qter→14q10::14q10→14qter)  46,XX,tas(5;22)(5qter→5pter→22qter→22pter),tas(14;15)(14qter→14pter→15pter→15qter)  46,XX,tas(14;15)(14qter→14pter→15pter→15qter)  46,XX,tas(17;18)(17qter→17pter→18pter→18qter) | 10 (16.66)  45, XX,-3  43, XX,-6,-11,-21  47,XX,+13  45, XX,-14  45,XX,-17  48,XX,+17,+22  45,XX,-21  45,X,-X[2]  93,XXXX,+1 | 6 (10.0)  46,XX,chrb(10)(q22.1),-22  45,XX,chtb(3)(q26.3),chtg(6)(p22.3),-18  44,XX,del(16) (pter→q23:),-19,-21  45,XX,-5,+dmin  45,XX,-8,tas(13;22)(13qter→  13pter→22pter→22qter)  45,XX,tas(15;21)(15qter→15pter→21pter→21qter),-19 |
| INF34A | 50  26 (52.0)  24 (48.0) | 20 (40.0)  46,X,chtg(X)(q26)  46,XX,del(16)(pter→q24:)  46,X,del(X)(pter→q28:)  45,XX,dic(14;22)(14qter→14p10::22p10→22qter),tas(15;22)(15qter→15pter→22pter→22qter)  46,XX,r(15)  45,XX,rob(14;22)(14qter→14q10::22q10→22qter)  46,XX,tas(12;19)(12qter→12pter→19pter→19qter),tas(14;21)(14qter→14pter→21pter→21qter),tas(21;22)(21qter→21pter→22pter→22qter)  46,XX,tas(13;13)(13qter→13pter→13pter→13qter)  46,XX,tas(13;14)(13qter→13pter→14pter→14qter)  46,XX,tas(13;15)(13qter→13pter→15pter→15qter)  46,XX,tas(13;18)(13qter→13pter→18pter→18qter)  46,XX,tas(14;13)(14qter→14qter→13pter→13qter)  46,XX,tas(14;15)(14qter→14pter→15pter→15qter)[2]  46,XX,tas(14;18)(14qter→14pter→18pter→18qter)  46,XX,tas(14;21)(14qter→14pter→21pter→21qter)  46,XX,tas(15;22)(15qter→15pter→22pter→22qter)  46,XX,tas(19;21)(19qter→19pter→21pter→21qter)[2]  46,XX,tr(14p;21p;21p) | 3 (6.0)  46, XX,-4,+5  42, XX,-5,-9,-15,-21  45,XX,-21 | 1 (2.0)  45,X,del(X)(q28),tas(14;21)(14qter→14pter→21pter→21qter),-18 |
| INF38A | 60  38 (63.3)  22 (36.7) | 10 (16.66)  46,XX,add(9)(q12q13)[5]  46,XX,chtb(7)(q12)  46,XX,chtg(13)(q33)  45,XX,rob(15;22)(15qter→15q12::22q12→22qter)  46,XX,tas(21;22)(21qter→21pter→22pter→22qter)[2] | 7 (11.66)  43,XX,-2,-5,-17  44,XX,-5,-19  44,XX,-15,-19  45,XX,-3  45, XX,-9  45,XX,-13  polyploidy | 5 (8.3)  44,XX,chtg (8)(q23)  44,XX,del(6)(pter→q25:),-11,-22  45,X,-X,del(12)(p12),chtg(7)(q11.2)  45,XX,-4,tas(14;17)(14qter→14pter→  17pter→17qter)  46,XX,tri(3;6),-14,+22 |
| INF39A | 50  32 (64.0)  18 (36.0) | 18 (36.0)  46,XX,add(9)(q12q13)  46,XX,dic(5),tas(21;22)(21qter→21pter→22pter→22qter)  46,X,fra(X)(q26),add(9)(q12q13)  45,XX,rob(15;22)(15qter→15q10::22q10→22qter)  46,XX,tas(13;15)(13qter→13pter→15pter→15qter)  46,XX,tas(13;21)(13qter→13pter→21pter→21qter)  46,XX,tas(13;22)(13qter→13pter→22pter→22qter)[2]  46,XX,tas(13;22)(13qter→13pter→22pter→22qter),tas(21;22)(21qter→21pter→22pter→22qter)  46,XX,tas(15;22)(15qter→15pter→22pter→22qter)[3]  46,XX,tas(15;22;22)(15qter→15pter→22pter→22pter→22qter)  46,XX,tas(21;21)(21qter→21pter→21pter→21qter)[2]  46,XX,tas(21;22)(21qter→21pter→22pter→22qter)[2]  46,XX,tas(22;22)(22qter→22pter→22pter→22qter) | 0 (0.0) | 0 (0.0) |
| INF44A | 50  37 (74.0)  14 (28.0) | 2 (4.0)  46,XX,tas(16;19)(16pter→16qter→19pter→19qter)  46,XX,tas(17;22)(17qter→17pter→22pter→22qter) | 7 (14.0)  45,XX,-4  43,XX,-6,-11,-13  45, XX, -10  42,XX,-10,-10,-14,-18  44,XX,-11,-19  45,XX,-16  45, XX,-22 | 5 (10.0)  45,X,del(X)(q23),-9,tas(13;22)(13qter → 13pter→22pter→22qter)  43,XX,-10,tas(13;15)(13qter→13pter→  15pter→15qter),-18,-21  45,XX,tas(13;21) (13qter→13pter→21qter→21pter),-22  44,XX,tas(13;22)(13qter→13pter→22pter→22qter),-20,-22  45,X,-X,tas(19;21)(19qter→19pter→ 21qter→21pter) |
| INF47A | 50  27 (54.0)  23 (46.0) | 15 (30.0)  46,XX,add(9)(q12q13)[6]  46,XX,chtg(2)(37.2)  46,XX,chtg(4)(q31)  46,XX,chtg(7)(q21)  46,XX,del(6)(q25:),del(15)(q11.1)  45,XX,dic(15;22)(15qter→15p10::22p10→22qter)  45,XX,rob(21;22)(21qter→21q10::22q10→22qter)  45,XX,tas(1;17)(1qter→1pter→17pter→17qter),add(9)(q12q13),-21  46,XX,tas(13;15)(13qter→13pter→  15pter→15qter)  46,XX,tas(15;21)(15qter→15pter→21pter→21qter) | 5 (10.0)  44,XX,-7,-19  45,XX,-10  47,XX,-10, +18,+21  47,XX,-12  45,XX,-17 | 3 (6.0)  47,XX,+5,tas(3;18)(3pter→3qter→18pter→18qter)  44,XX,-7,add(9) (q12q13)  46,XX,tas(13;21)(13qter→13pter→21pter→21qter)/ |
| INF53A | 50  41 (82.0)  9 (18.0) | 3 (6.0)  46,XX,tas(11;21)(11pter→11qter→21pter→21qter)[2]  46,XX,tas(13;14)(13qter→13pter→14pter→14qter),tas(19;22)(19pter→19qter→22pter→22qter) | 4 (8.0)  43, XX,-2,-10,-15  46,XX, +3,-8  44,XX,-20,-22  45,XX,-22 | 2 (4.0)  42,XX,del(6)(q22),-8,-19,-20,-21  46,XX,dic(8;?)(q24.3;?),-15 |
| INF60A | 50  46 (92.0)  4 (8.0) | 0 (0.0) | 3 (6.0)  45,XX,-11  45,XX,-20  45,XX,-21 | 1 (2.0)  44,X,chtb(X)(q22),dic(?3)t(3;?7)(3pter→3q21::?7q22→7pter),-7,-12 |
| INF65A | 50  40 (80.0)  10 (10.0) | 5 (10.0)  46,XX,+ace  46,XX,tas(13;14)(13qter→13pter→14pter→14qter)  46,XX,tas(13;22)(13qter→13pter→22pter→22qter)  46,XX,tas(15;22)(15qter→15pter→22pter→22qter)  46,XX,tas(21;22)(21qter→21pter→22pter→22qter) | 2 (4.0)  45,XX, -17  42,XX,-17,-17,-18,-18 | 3 (6.0)  42,X,chtb(X)(q22),-4,-14,-15,-16  45,XX,-9,tas(14;22)(14qter→14pter→ 22 pter→22qter)  44,XX,tas(13;15)(13qter→13pter→15pter→15qter),tas(14;21;16)(14qter→14pter→21pter→21qter→16qter→16pter),-17,-22 |
| INF66A | 50  38 (76.0)  12 (24.0) | 3 (6.0)  46,XX,rob(13;15)(13qter→13p10::15p10→15qter)  46,XX,tas(1;10)(1qter→1pter→10pter→10qter)  46,XX,tas(13;13)(13qter→13pter→13pter→13qter) | 7 (14.0)  46, XX,-4, +10  46, XX,-9, +10  41, XX, -15,-16,-18,-18,-20  45,XX,-18  43,XX,-20,-21,-22  45, XX,-22,+ace  45,X,-X | 2 (4.0)  40,XX,-7,-8,-15,-16,-20,-22,+ace  44,XX, del(6)(q22.1),-13,-19 |
| INF87A | 50  33 (66.0)  17 (34.0) | 12 (24.0)  46,XX,add(9)(q12q13)[3]  46,XX,chrb(6)(q16.3),tas(15;22)(15qter→15pter→22pter→22qter)  46,X,del(X)(q27)  46,XX,tas(6;22)(6qter→6pter→22pter→22qter),tas(16;21)(16pter→16qter→21pter)  46,XX,tas(11;18)(11qter→11pter→18qter→18pter  46,XX,tas(14;15)(14qter→14pter→15pter→15qter),tas(21;22)(21qter→21pter→22pter→22qter)  46,XX,tas(15;22)(15qter→15pter→22pter→22qter)  46,XX,tas(15;22)(15qter→15pter→22qter→22pter)  46,XX,tas(19;21)(19qter→19pter→21pter→21qter)  46,tas(21;22)(21qter→21pter→22pter→22qter) | 3 (6.0)  44,XX,+4,-15,-22  44,XX,-5,-8,-10, +12  45, XX, -8 | 2 (4.0)  46,XX,-13,-15,+ace,+ace  42,X,-X,-6,-17, -18,+ace |
| INF92A | 50  40 (80.0)  10 (20.0) | 6 (12.0)  46,X,del(X)(q24:),tas(14;21)(14qter→14pter→21pter→21qter)  46,XX,tas(4;21)(4qter→4pter→21pter→21qter)  46,XX,tas(5;17)(5qter→5pter→17pter→17qter),tas(13;13)(13qter→13pter→13pter→13qter)  45,XX,tas(11;15)(11qter→11p15.4::15p11.2→15qter)  46,XX,tas(17;21)(17qter→17pter→21qter→21pter)  46,XX,tas(18;22)(18qter→18pter→22pter→22qter) | 4 (8.0)  46,XX,-1,+8, +17  45,XX,-10  45,XX,-15  46,X,-X,-1,+8,+17 | 0 (0.0) |
| INF98A | 50  31 (62.0)  19 (38.0) | 16 (32.0)  46,XX,add(9)(q12q13)[6]  46,XX,chtb(4)(q25)  46,XX,chtg(2)(q33),add(9)(q12q13)  46,X,del(X)(q26)  46,XX,16qh+  46,XX,tas(3;14)(3pter→3qter→14pter→14qter)  46,XX,tas(13;22)(13qter→13pter→22pter→22qter)  46,XX,tas(14;15)(14qter→14pter→15pter→15qter)  46,XX,tas(14;22)(14qter→14pter→22pter→22qter)  46,XX,tas(21;22)(21qter→21pter→22pter→22qter)[2]  46,XX,tas(15;22)(15qter→15pter→22pter→22qter) | 2 (4.0)  46, XX,+3,-4  45,XX,-8 | 1 (2.0)  51,XX,+1,+6,+7,-8,+11,tas(13;15)(13qter →13pter→15pter→15qter),+14,+21 |
| INF109A | 50  40 (80.0)  10 (20.0) | 9 (18.0)  46,X,del(X)(q26),tas(21;22)(21qter→21pter→22pter→22qter)  46,XX,dic(X;10)(Xqter→Xpter::10pter→10qter)  pcd46,XX  46,XX,tas(13;15)(13qter→13pter→15pter→15qter)[2]  46,XX,tas(13;21)(13qter→13pter→21pter→21qter)[2]  46,XX,tas(13;22)(13qter→13pter→22pter→22qter)  46,XX,tas(21;21)(21qter→21pter→21pter→21qter) | 1 (2.0)  45,XX,-17 | 0 (0.0) |

**Supplementary Table 3(c): Representative karyotypes for males with secondary infertility harbouring the structural aberrations with increased frequency in infertile patients.**

| **Males with Secondary Infertility** | **TMS**  **Normal (%)**  **TAM (%)** | **Representative Karyotypes** | | |
| --- | --- | --- | --- | --- |
|  |  | **TMSA (%)** | **TMNA (%)** | **TM(SA+NA) (%)** |
| INF29B | 100  46 (46.0)  54 (54.0) | 38 (38.0)  46,XX,add(9)(q12q13)[23]  46,XY,add(9)(q12q13),chrb(9)(q12q13)  46,XY,add(9)(q12q13),chrb(14)(q24.3)  46,XY,add(9)(q12q13),del(16)(pter→q23:)  46,XY,add(9)(q12q13),del(18)(q22)  46,XY,add(9)(q12q13),dic(15;17)(15qter→15p10::17p10→17qter)  46,XY,add(9)(q12q13),tas(9;17)(9pter→9qter→17pter→17qter),tas(13;22)(13qter→13pter→22pter→22qter),tas(14;21)(14qter→14pter→21pter→21qter)  46,XY,add(9)(q12q13),tas(14;15)(14qter→14pter→15pter→15qter)  45,XY,chrb(3)(q21)  46,XY,chtb(17)(q21)  46,XY,dic(8;21)(8pter→8q24.2::21p10→21qter),tas(20;22)(20pter→20qter→22qter→22pter)  46,XY,tas(1;11)(1qter→1qter→11qter→11pter),dic(5;22)(5qter→5p11.2::22p10→22pter)  46,XY,tas(6;20)(6pter→6qter→20pter→20qter)  46,XY,tas(13;18)(1qpter→13p)ter→18pter→18qter)  46,XY,tas(14:15)(14qter→14pter→15pter→15qter)  46,X,tas(Y;13)(Yqter→Ypter→13pter→13qter) | 5 (5.0)  43,XY,-3,-11,-20  45,XY,-11  45,XY,-16  45,XY,-21  43,X,-Y,-3,-22 | 11 (11.0)  43,XY,-3, add(9) (q 12q13),-11,-20  43,XY,-2,-3, add(9) (q12q 13),-13  45,XY, add(9) (q 12q13),-21  46,XY,chrb(8)(q21.1), add(9)(q12q13)  40,XY,chtb(1)(q22),-6,add(9)(q12q13), chtb(11)(q13.1) -12,-20,-21,-21,-22  45,XY, del (6)(p24)  42,XY,-3,del(13)  (p11.2),tas(11;21)(11qter→11pter→21qter→21pter),-16,-16,-19  44,X,t(Y;22)(Yqter→Yq10::22q 10;22qter),tas(2;10;18;12)(2qter→  2cent→10pter→10pter→18pter→12pter→12qter),-22  46, XY, tas(5; 9;7) (5qter→ 5 pter→ 9pter →7pter→7qter)  45,XY,-16,tas(5;15)(5qter→5pter→  15pter→15qter)  44,XY,-12,tas(15;21)(15qter→15 pter→21pter→21qter),-20 |
| INF68B | 50  32 (62.0)  18 (36.0) | 15 (30.0)  46,XY,del(1)(q22.24),add(9)(q12q13)  46,XY,del(16)(q22)/46,XY,del(22)(p11.2)  46,XY,add(9)(q12q13),tas(14;15)(14qter→14pter→15pter→15qter)  46,XY,add(9)(q12q13),tas(20;20)(20qter→20pter→20pter→20qter)  46,XY,chrb(1)(q22.24),add(9)(q12q13)  46,XY,chtb(4)(q26)  46,XY,chtb(5)(q22),tas(20;21)(20pter→20qter→21pter→21qter)[2]  46,XY,chtb(6)(p12)  46,XY,tas(5;22)(5pter→5qter→22pter→22qter)  46,XY,tas(11;21)(11qter→11pter→21pter→21qter)  46,XY,tas(14;15)(14qter→14pter→15pter→15qter)  46,XY,tas(14;21)(14qter→14pter→21pter→21qter)  46,XY,tas(15;22)(15pter→15qter→22qter→22pter)  46,XY,tas(17;21)(17qter→17pter→21pter→21qter) | 3 (6.0)  45,XY,-17  44,XY,-17,-20  45,XY,-22 | 0 (0.0) |

**Supplementary Table 3(d): Representative karyotypes for females with secondary infertility harbouring the structural aberrations with increased frequency in infertile patients.**

| **Females with Secondary Infertility** | **TMS**  **Normal (%)**  **TAM (%)** | **Representative Karyotypes** | | |
| --- | --- | --- | --- | --- |
|  |  | **TMSA (%)** | **TMNA (%)** | **TM(SA+NA) (%)** |
| INF59A | 50  43 (86.0)  7 (14.0) | 5 (10.0)  46,XX,chtb(17)(q21)  45,XX,del(6)(q26),add(9)(q12q13)  46,XX,dic(7)  46,XX,tas(21;21)(21qter→21pter→21pter→21qter)  47,XX,+X,tas(22;22)(22qter→22pter→22pter→22qter) | 1 (2.0)  45,XX,-10 | 1 (2.0)  44,XX,rob(13;21)(13qter→13q10::21q10→21qter),-21 |
| INF67A | 50  17 (34.0)  33 (66.0) | 23 (46.0)  46,XX,tas(13;21)(13qter→13pter→21pter→21qter)  46,XX,add(9)(q12q13)[3]  46,XX,chrb(9)(q13),tas(13;15)(13qter→13pter→15pter→15qter)  46,XX,chtb(1)(q31),tas(8;21)(8qter→8pter→21qter→21pter),tas(11;12)(11qter→11pter→12pter→12qter),tas(13;21)(13qter→13pter→21pter→21qter)  46,X,del(X)(q22),tas(1;12)(1pter→1qter→12qter→12pter),tas(19;21)(19pter→19qter→21pter→21qter)  46,X,del(X)(q25)  45,XX,dic(13;14)(13qter→13p10::14p10→14qter)  45,XX,dic(13;21)(13qter→13p10::21p10→21qter)  45,XX,rob(13;14)(13qter→13q10::14q10→14qter)  45,rob(14;15)(14qter→14p10::15p10→15qter)  45,XX,rob(22;22)(22qter→22p10::22p10→22qter)  46,XX,tas(1;12)(1pter→1q43→12q24.3→12qter),tas(19;21)(19pter→19qter→21pter→21qter  46,XX,tas(4;13)(4pter→4q34→13q33→13pter)  46,XX,tas(7;17)(7pter→7qter→17qter→17pter)  46,XX,tas(13;13)(13qter→13pter→13pter→13qter)  46,XX,tas(13;14;17)(13qter→13pter→14pter→14qter→17qter→17pter)  46,XX,tas(13;15)(13qter→13p13::15p13→15qter)  46,XX,tas(13;21)(13qter→13pter→21pter→21qter)  46,XX,tas(14;14)(14qter→14pter→14pter→14qter)[2]  46,XX,tas(14;21)(14qter→14pter→21pter→21qter) | 6 (12.0)  45,XX,-13  45,XX,-14  45,XX,-15  43,X,-X,-8,-16  45,XX,-22[2] | 4 (8.0)  45,XX,-14,+ace  45,X,del(X)(q25),del(7)(q33)  45,XX,tr(2;15;21),-21  43,XX,-7,add(9)(q12q13),-12,tas(14;22)(14qter→14pter→22pter→22qter),-16 |
| INF68A | 50  28 (56.0)  22 (44.0) | 10 (20.0)  46,X,del(X)(q23)[2]  46,X,del(X)(q27),tas(14;21)(14qter→14pter→21pter→21qter)  46,XX,dic(14;22)(22qter→22p12::14p12→14qter)  46,XX,r(21)  45,XX,rob(21;22)(21qter→21q10::22q10→22qter),tas(13;15)(13qter→13pter→15pter→15qter)  46,XX,tas(13;14)(13qter→13pter→14pter→14qter)  46,XX.tas(13;21)(13qter→13pter→21pter→21qter),tas(14;21)(14qter→14pter→21pter→21qter)  46,XX,tas(14;15)(14pter→14qter→15qter→15pter)  46,XX,tas(14;21)(14qter→14pter→21pter→21qter) | 2 (4.0)  42,XX,-2,-3,-4,-19  44,XX,-15,-22 | 10 (20.0)  47,XX,+2, tas(13;15)(13qter→13pter→15pter→15qter),tas(21;22)(21qter→21pter→22pter→22qter)  45,XX,-15,-22,+ace  45,XX,-8,add(9)(q12q13)  44,X,del(X)(q27),-6,-17  45,XX,rob(15;22)(15qter →15q10::22p10→22qter)  46,XX,tas(7;12)(7pter→7qter→12qter→12pter)(14;21)(14qter→14pter→21pter→21qter),-17  45,XX,tas(13;14;21)(13qter  →13pter→14pter→21pter→21qter)-19  45,XX,-16,tas(13;15) (13qter→  13pter→15pter→15qter)  45,XX,tas(13;15)(13qter→13pter→15pter→15qter),-16  44,X,-10,tas(14;13)(14qter→  14pter→13pter→13qter) |
| INF77A | 50  39 (78.0)  11 (22.0) | 3 (6.0)  46,X,del(X)(q25)  45,XX,rob(15;22)(15qter→15q10::22q10→22qter) | 5 (10.0)  46, XX,+5,-10  46,XX, +6,-9  44,XX,-7,-14  43,XX,-8,-8,-15  45,XX,-11 | 3 (6.0)  46,XX,-11,+ace  46,X,del(X)(q27),+5,-6,tas(13;14; 21)(14qter→14pter→13pter→21pter→21qter/45,X,del(X)(q25),-11  46,XX,+5,-10, tas(13;14;21) (14qter→  14pter→13pter→21pter→21qter) |

**Supplementary Table 3(e): Representative karyotypes for male controls age-matched with primary infertility patients harbouring the structural aberrations with increased frequency in infertile patients.**

| **Male controls for Primary Infertility** | **TMS**  **Normal (%)**  **TAM (%)** | **Representative Karyotypes** | | |
| --- | --- | --- | --- | --- |
|  |  | **TMSA (%)** | **TMNA (%)** | **TM(SA+NA) (%)** |
| C283 | 50  44 (88.0)  6 (12.0) | 3 (6.0)  46,XY,chrb(17)(q21.3)  45,XY,rob(15;21)(15qter→15q10::21q10→21qter)  46,XY,tas(21;22)(21qter→21pter→22pter→22qter) | 3 (6.0)  42,XY,-5,-9,-11,-12  45, XY,-6  45,XY,-11  41,XY,-11,-12,-13,-15,-19  45,XY,-15 | 0 (0.0) |
| UA86 | 100  70 (70.0)  30 (30.0) | 10 (10.0)  46,XY,chrb(17)(q21)  46,XY,tas(13;21) (13qter→13pter→ 21pter →21qter) | 20 (20.0)  44,XY,-15,-21  44,X,-Y,-8  45,XY,-12 | 0 (0.0) |
| C107 | 101  93 (92.08)  8 (7.92) | 4 (3.96)  46,XY,chrb(5)(q22)  46,XY,tas(Y;21) (Yqter→Ypter→21pter →21qter) | 4 (3.96)  45,XY,-8  45,XY,-12 | 0 (0.0) |
| UA76 | 100  73 (73.0)  27 (27.0) | 15 (15.0)  46,XY,chtb(7)(q22),add(9)(q13)  46,XY,chtb(10)(q24)  46,XY,chtg(1)(p22) | 11 (11.0)  44,XY,-3,-9  45,XY,-7  45,XY,-12 | 1 (1.0)  42,XY,-12,-15,rob(21;22) (21qter→21q10::22q10→ 22qter),-22 |
| UA65 | 100  78 (78.0)  22 (22.0) | 8 (8.0)  46,XY,del(5)(pter→5q23:),tas(5;9;5)(5pter→5q23→9pter→9qter→5pter→5qter),+ace?(5)(:q23→qter)  46,XY,tas(13;21) (13qter →13pter →21pter→21qter)  46,XY, tr(14;15;22)(p13; p13;p13) | 12 (12.0)  46,XY,+9,-17  45,XY,-13  46,X, -Y,+20 | 2 (2.0)  45,XY,+4,-7,-8,chrb(12)(q24.1),  tas(13;15)(13qter→13pter→15pter→ 15qter),+der(16)(pter→q2::?::qter) |

**Supplementary Table 3(f): Representative karyotypes for female controls age-matched with primary infertility patients harbouring the structural aberrations with increased frequency in infertile patients**

| **Female controls for Primary Infertility** | **TMS**  **Normal (%)**  **TAM (%)** | **Representative Karyotypes** | | |
| --- | --- | --- | --- | --- |
|  |  | **TMSA (%)** | **TMNA (%)** | **TM(SA+NA) (%)** |
| UA19 | 106  86 (81.0)  20 (19.0) | 11 (10.4)  46,XX,chtb(9)(q12)  46,XX,chtg(6)(q21) | 7 (6.6)  43, XX,-3,-9,-10  44,XX,-4,-5  41,XX,-8,-9,-10,-15,-20  45,XX,-12  45,XX,-20  45, XX,-22 | 2 (1.9)  44,XX,-2,-5,+ace,+ace |
| M54 | 50  43 (86.0)  7 (14.0) | 1 (2.0)  46,X,del(X)(q26) | 6 (12.0)  45,XX,-8  44,XX,-9,-20  45,XX,-10  45,XX,-16  45,XX,-18  45,XX,-22 | 0 (0.0) |
| C166 | 100  75 (75.0)  25 (25.0) | 15 (15.0)  46,XX,chrb(16)(q21)  46,XX,tas(4;17)(4qter→4pter→17pter→17qter)  46,XX,tas(15;22) (15qter→15pter→22pter →22qter) | 7 (7.0)  48,XX,+3,+5  45,XX,-7  45,XX,-17 | 3 (3.0)  45,X,-X,chrb(16)(q22) |

**Supplementary Table 3(g): Representative karyotypes for male controls age-matched with secondary infertility patients harbouring the structural aberrations with increased frequency in infertile patients**

| **Male controls for Secondary Infertility** | **TMS**  **Normal (%)**  **TAM (%)** | **Representative Karyotypes** | | |
| --- | --- | --- | --- | --- |
|  |  | **TMSA (%)** | **TMNA (%)** | **TM(SA+NA) (%)** |
| C233 | 50  47 (94.0)  3 (6.0) | 1 (2.0)  46,XY,chtb(10)(q22) | 1 (2.0)  39,X,-Y,-1,-3,-7,-11,  -15,-19 | 1 (2.0)  45,XY,-13,tas(15;15)(15qter →15pter→15pter→15qter) |

**Supplementary Table 3(h): Representative karyotypes for female controls age-matched with secondary infertility patients harbouring the structural aberrations with increased frequency in infertile patients**

| **Female controls for Secondary Infertility** | **TMS**  **Normal (%)**  **TAM (%)** | **Representative Karyotypes** | | |
| --- | --- | --- | --- | --- |
|  |  | **TMSA (%)** | **TMNA (%)** | **TM(SA+NA) (%)** |
| C135 | 100  76 (76.0)  24 (24.0) | 6 (6.0)  46,XX,del(6) (q23q24)  46,XX,qr(13;14;15;22)  45,XX,rob(13;13)(q10;q10)  45,XX,rob(21;22)(q10;q10)  45,XX,rob(22;22)(q10;q10) | 17 (17.0)  44, XX,-4,-17  39,X,-X,-8,-12,-13,  -20, -21,-22  45,XX, -9,+11,-17  45, XX,-13  45,XX,-18 | 1 (1.0)  47,XX,+2,terdel(3)(p25) |
| C169 | 100  82 (82.0)  18 (18.0) | 11 (11.0)  46,XX,del(X)(pter→q25:),tas(19;21)(19pter→19qter→21pter→21qter)  46,XX,tas (15;21) (15qter→15pter→21pter→ 21qter) | 6 (6.0)  46,XX,-8,+22  47,XX, +12  45,XX,-22  45,X,-X | 1 (1.0)  45,XX,-8,tas(15;22)(15qter →15pter→22pter→22qter) |

**Supplementary Table 4: Compilation of a list of published reports on the roles of the genes within the male infertility network in the maintenance of male fertility.**

| **Region** | **S. No.** | **Gene (NCBI Gene ID)** | **Official Name** | **Cytogenetic location** | **Exons** | **Literature related to role in male fertility** | **References** |
| --- | --- | --- | --- | --- | --- | --- | --- |
| 5q2 | 1 | *APC* (324)* | Adenomatous polyposis coli | 5q22.2 | 20 | Progressive degeneration of seminiferous tubules observed in male mice with conditional knockout of *APC* | **[29]** |
|  | 2 | *FER* (2241)* | FER tyrosine kinase | 5q21.3 | 33 | The truncated testicular isoform of FER, known as FerT (expressed in rat pachytene spermatocytes and round spermatids) may play a role in acrosome development | **[30]** |
|  | 3 | *HINT1* (3094) | Histidine triad nucleotide binding protein 1 | 5q23.3 | 5 | Expression of *HINT1* was downregulated in asthenozoospermic males compared to normozoospermic controls | **[31]** |
| 10q2 | 4 | *ADAMTS14* (140766) | ADAM metallopeptidase with thrombospondin type 1 motif 14 | 10q22.1 | 26 | A significant decrease in sperm DNA methylation level was detected in an intronic CpG site within *ADAMTS14* in sub-fertile males compared to fertile males | **[32]** |
|  | 5 | *BUB3* (9184)* | Budding uninhibited by benzimidazole 3 | 10q26.13 | 8 | *Zmym3* KO mice were infertile with spermatogenesis arrested at MI. The KO mice had a two-fold increased number of *BUB3+* MI spermatocytes compared to wildtype mice | **[33]** |
|  | 6 | *COL13A1* (1305) | Collagen type XIII alpha 1 chain | 10q22.1 | 43 | Downregulationof *COL13A1* expression in Klinefelter syndrome patients with hypospermatogenesis | **[34]** |
|  | 7 | *CTBP2* (1488) | C-terminal binding protein 2 | 10q26.13 | 26 | *CTBP2* was among several significantly expressed transcription factors in males with NOA compared with vasectomy controls | **[35]** |
|  | 8 | *DDX21* (9188) | DExD-box helicase 21 | 10q22.1 | 16 | *DDX3Y* facilitates male germline development by activating the expression of several genes involved in RNA metabolism such as *DDX21*, *KHDRBS3*, *SF3A1*, *RAB39A* and *RPRD2* | **[36]** |
|  | 9 | *EBF3* (253738) | EBF transcription factor 3 | 10q26.3 | 19 | *EBF3* showed significant differences in DNA methylation in oligozoospermic/oligoasthenozoospermic males compared with normozoospermic controls | **[37]** |
|  | 10 | *FANK1**(92565) | Fibronectin type III and ankyrin repeat domains 1 | 10q26.2 | 13 | Increased levels of apoptosis resulted in oligospermia in *Fank1*-knockdown mice | **[38]** |
|  |  |  |  |  |  | No alterations in spermatogenesis in *Fank1* KO mice | **[39]** |
|  | 11 | *GRK5* (2869) | G protein-coupled receptor kinase 5 | 10q26.11 | 18 | *GRK5* was upregulated in maturation arrest azoospermia and teratospermia | **[40]** |
|  | 12 | *INPP5F* (22876)* | Inositol polyphosphate-5-phosphatase F | 10q26.11 | 25 | *Inpp5b-/-* mice exhibited reduced fertility | **[41]** |
|  | 13 | *NEUROG3* (50674) | Neurogenin 3 | 10q22.1 | 2 | *STAT3-*regulated *NEUROG3* expression mediates the differentiation of SSCs and progenitor spermatogonia in mammals | **[42]** |
|  | 14 | *PLAU* (5328)* | Plasminogen activator, urokinase | 10q22.2 | 12 | Downregulation of *PLAU* (also known as *uPA*) possibly decreases the fertility of male mice by reducing sperm motility | **[43]** |
|  | 15 | *TUBGCP2* (10844) | Tubulin gamma complex associated protein 2 | 10q26.3 | 20 | Differential protein phosphorylation of TUBGCP2 (also known as GCP2) detected in asthenozoospermic males compared with fertile controls | **[44]** |
|  | 16 | *VCL* (7414) | Vinculin | 10q22.2 | 22 | The association of Vinculin, Focal Adhesion Kinase, Paxillin and Talin with the Focal Adhesion Complex in mammalian spermatozoa is necessary to maintain acrosome integrity | **[45]** |
|  | 17 | *ZMIZ1* (57178) | Zinc finger MIZ-type containing 1 | 10q22.3 | 30 | miR-29 reported to significantly downregulate *ZMIZ1* expression at different meiotic stages in developing male germ cell lines | **[46]** |
|  | 18 | *ZNF503* (84858) | Zinc finger protein 503 | 10q22.2 | 5 | *ZNF503* was significantly downregulated in 11.5 dpc male somatic gonad cells | **[47]** |
|  | 19 | *ZSWIM8* (23053) | Zinc finger SWIM-type containing 8 | 10q22.2 | 28 | *ZSWIM8* was predicted to be a target gene for hsa-miR-6723-5p, a microRNA which was upregulated in testicular tissues of NOA patients with unsuccessful sperm retrieval compared with NOA patients with successful sperm retrieval | **[48]** |
| 17q2 | 20 | *ACLY* (47) | ATP citrate lyase | 17q21.2 | 30 | ACLY exhibited increased protein phosphorylation in low motility sperm samples compared to high motility sperm samples, both isolated from normozoospermic males | **[49]** |
|  | 21 | *BECN1* (8678) | Beclin 1 | 17q21.31 | 12 | The sperm of infertile patients had a significantly increased expression of Beclin1 mRNA and protein compared with healthy controls | **[50]** |
|  | 22 | *BRCA1* (672)* | Breast cancer type 1 susceptibility gene | 17q21.31 | 24 | Disruption of interaction of *BRCA1* and *PALB2* results in fertility defects in male mice model | **[51]** |
|  | 23 | *CBX1* (10951)* | Chromobox 1 | 17q21.32 | 6 | Mice with testes-specific *Cbx1* (also known as *HP1β*) cKO exhibit impaired spermatogenesis | **[52]** |
|  | 24 | *CCR7* (1236)* | C-C motif chemokine receptor 7 | 17q21.2 | 5 | *CCR7* mRNA levels were upregulated in dendritic cells isolated from testes of rats with EAO compared with control rats | **[53]** |
|  | 25 | ***CDC27***(996) | Cell division cycle 27 | 17q21.32 | 20 | *CDC27* was significantly downregulated in asthenozoospermic individuals compared with normozoospermic individuals | **[20]** |
|  | 26 | *CDC6* (990) | Cell division cycle 6 | 17q21.2 | 13 | *CDC6* was significantly upregulated in seminal plasma of males with OA compared with males with NOA | **[54]** |
|  | 27 | *CDK5RAP3* (80279)* | CDK5 regulatory subunit associated protein 3 | 17q21.32 | 16 | The expression of *Cdk5rap3* was increased 1.57-fold in mice XY germ cells at 14.5 dpc compared with 12.5 dpc | **[55]** |
|  | 28 | *COL1A1* (1277)* | Collagen type I alpha 1 chain | 17q21.33 | 51 | *Col1a1* mediates the detachment and migration of germ cells in male mice model | **[56]** |
|  | 29 | *CSF3* (1440) | Colony stimulating factor 3 | 17q21.1 | 4 | Seminal levels of CSF3 were significantly higher in males with high seminal ROS levels compared with normal controls. | **[57]** |
|  | 30 | *EME1* (146956) | Essential meiotic structure-specific endonuclease 1 | 17q21.33 | 10 | *EME1* expression was upregulated in oligozoospermic males compared with normozoospermic males | **[58]** |
|  | 31 | *ETV4* (2118)* | ETS variant transcription factor 4 | 17q21.31 | 14 | *Etv4*-/- male mice demonstrated sexual dysfunction and did not mate with female mice despite no observable defects in reproductive organs and sperm activity | **[59]** |
|  | 32 | *FZD2* (2535) | Frizzled class receptor 2 | 17q21.31 | 1 | *FZD1*, *FZD2* and *FZD5* were upregulated in the IHCE1 cell line compared with the FHCE1 cell line | **[60]** |
|  | 33 | *GFAP* (2670)* | Glial fibrillary acidic protein | 17q21.31 | 10 | The selective depletion of GFAP-expressing tanycytes in GFAP-Tk mice using ganciclovir resulted in a marked reduction in testosterone levels and testicular weight, seminiferous tubule vacuolization and loss of spermatogenesis | **[61]** |
|  | 34 | *HOXB1* (3211) | Homeobox B1 | 17q21.32 | 2 | *HOXB1* showed significant methylation differences in spermatozoal DNA from infertile men compared to fertile men | **[37]** |
|  | 35 | *HOXB2* (3212) | Homeobox B2 | 17q21.32 | 3 | High expression of *HOXB2* in T1LCs | **[62]** |
|  | 36 | *HOXB4* (3214) | Homeobox B4 | 17q21.32 | 2 | High expression of *HOXB4* in T1LCs with weak or no expression in oogonia like cells | **[62]** |
|  | 37 | *HOXB5* (3215) | Homeobox B5 | 17q21.32 | 2 | High expression of *HOXB5* in T1LCs | **[62]** |
|  | 38 | *HOXB6* (3216) | Homeobox B6 | 17q21.32 | 5 | Loss of methylation at *HOXB6* loci in sperm reported to be associated with male age and reproductive outcome in infertility treatment. | **[63]** |
|  | 39 | *HOXB7* (3217) | Homeobox B7 | 17q21.32 | 2 | *HOXB7* was predicted as a target gene targeted by microRNAs upregulated in male infertility patients | **[64]** |
|  | 40 | *HOXB8* (3218) | Homeobox B8 | 17q21.32 | 5 | High expression of *HOXB8* in T1LCs | **[62]** |
|  | 41 | *HOXB9* (3219) | Homeobox B9 | 17q21.32 | 2 | High expression of *HOXB9* in T1LCs | **[62]** |
|  | 42 | *ITGA3* (3675)* | Integrin subunit alpha 3 | 17q21.33 | 26 | *Itga3* and several other proteins involved in cell adhesion and cell-cell junction formation were upregulated in the spermatocytes of infertile *Dicer1* KO mice | **[65]** |
|  | 43 | *JUP* (3728)* | Junction plakoglobin | 17q21.2 | 19 | The downregulation of *Dsg1b* and *Jup* in desmosome-like junctions might explain the destabilization and separation of germ cells in *Crem* knockout mice | **[66]** |
|  | 44 | *KAT2A* (2648)* | Lysine acetyltransferase 2A | 17q21.2 | 18 | *Kat2a* (also known as *Gcn5*) mediates histone acetylation and nucleosome dynamics in spermiogenesis | **[67]** |
|  | 45 | *KPNB1* (3837) | Karyopherin subunit beta 1 | 17q21.32 | 23 | *KPNB1* participates in proteostasis in male germ cells | **[68]** |
|  | 46 | *KRT14* (3861) | Keratin 14 | 17q21.2 | 8 | *KRT14* expression was significantly downregulated in males with NOA compared with vasectomy controls | **[35]** |
|  | 47 | *KRT16* (3868)* | Keratin 16 | 17q21.2 | 8 | A reduction in the levels of Krt16 protein was observed in the epididymis of rats exposed to cigarette smoke | **[69]** |
|  | 48 | *KRT17* (3872) | Keratin 17 | 17q21.2 | 8 | *KRT17* expression was significantly downregulated in males with NOA compared with vasectomy controls | **[35]** |
|  | 49 | *KRT19* (3880)* | Keratin 19 | 17q21.2 | 6 | *Krt19* mutants have altered reproductive phenotypes in male mice | **[70]** |
|  | 50 | *MAP3K14* (9020) | Mitogen-activated protein kinase kinasekinase 14 | 17q21.31 | 17 | *MAP3K14* showed altered expression in testes of males with azoospermic non-mosaic Klinefelter Syndrome compared with normal control testes | **[71]** |
|  | 51 | *NME1* (4830) | Non-metastatic cells 1 | 17q21.33 | 6 | NME1 protein levels were significantly increased in males with high sperm DNA fragmentation compared with males with low sperm DNA fragmentation | **[72]** |
|  | 52 | *PSMC3IP* (29893) | PSMC3 interacting protein | 17q21.2 | 9 | An azoospermic male from a consanguineous Yemeni family was found to be a carrier of a homozygous *PSCM3IP* stop-gain mutation located in exon 6. The same mutation was identified in four female siblings diagnosed with POI | **[73]** |
|  | 53 | ***PSMD3***(5709) | Proteasome 26S subunit, non-ATPase 3 | 17q21.1 | 12 | *PSMD3* was significantly downregulated in asthenozoospermic individuals compared with normozoospermic individuals | **[20]** |
|  | 54 | ***PSME3***(10197)* | Proteasome activator subunit 3 | 17q21.31 | 12 | Complete infertility in *Psme3*/*Psme4*dKO male mice due to significant reduction in sperm motility and proteasome activity | **[18]** |
|  |  |  |  |  |  | Subfertility in male mice with *Psme3* deficiency due to decrease in concentration and activity of sperm | **[19]** |
|  | 55 | *RARA* (5914)* | Retinoic acid receptor alpha | 17q21.2 | 17 | Spermatogenesis disrupted in *Rara* -/- male mice | **[74]** |
|  | 56 | *STAT3* (6774)* | Signal transducer and activator of transcription 3 | 17q21.2 | 24 | *STAT3^N-/-^* mutation led to the development of hypogonadism and infertility in both male and female mice | **[75]** |
|  | 57 | *THRA* (7067) | Thyroid hormone receptor alpha | 17q21.1 | 11 | *THRA* appears to play a critical role in testicular development and function | **[76]** |
|  | 58 | *TOP2A* (7153) | DNA topoisomerase II alpha | 17q21.2 | 36 | *TOP2A* was significantly downregulated in testicular tissues of SCOS males compared to controls with obstructive azoospermia | **[77]** |
|  | 59 | *TUBG1* (7283) | Tubulin gamma 1 | 17q21.2 | 11 | TUBG1 was significantly downregulated in idiopathic azoospermia. | **[78]** |
|  | 60 | *TUBG2* (27175) | Tubulin gamma 2 | 17q21.2 | 12 | *TUBG2* expression was reduced in asthenozoospermic males compared with fertile controls | **[44]** |
|  | 61 | *WNT3* (7473) | Wnt family member 3 | 17q21.31-q21.32 | 5 | Defective expression of *Wnt3* by Sertoli cells affects fertility in males | **[79]** |
|  | 62 | *WNT9B* (7484)* | Wnt family member 9B | 17q21.32 | 6 | Epididymis and vas deferens were absent in the testes of *Wnt9b*-/- mice at birth | **[80]** |

Hub genes are highlighted in bold, * - Reports including animal models, cKO: Conditional knockout; Crem: cAMP responsive element modulator; Dicer1: Dicer1, ribonuclease III; dKO: double knockout; dpc: days past coitum; Dsg1b: Desmoglein 1 beta; EAO: Experimental autoimmune orchitis; FHCE1: Fertile human caput epididymal cell line 1; FZD1: Frizzled class receptor 1; FZD5: Frizzled class receptor 5; GFAP-Tk: Glial fibrillary acidic protein-thymidine kinase; IHCE1: Infertile human caput epididymal cell line 1; KHDRBS3: KH RNA binding domain containing, signal transduction associated 3; KO: Knockout; MI: Meiosis I; NOA: Non-obstructive azoospermia; OA: Obstructive azoospermia; PALB2: Partner and localizer of BRCA2; POI: Primary ovarian insufficiency; RAB39A: RAB39A, member RAS oncogene family; ROS: Reactive oxygen species; RPRD2: Regulation of nuclear pre-mRNA domain containing 2; SCOS: Sertoli-Cell-Only Syndrome; SF3A1: Splicing factor 3a subunit 1; SSCs: Spermatogonial cells; STAT3: Signal transducer and activator of transcription 3; T1LCs: T1 prospermatogonia-like cells.

**Supplementary Table 5: Compilation of a list of published reports on the roles of the genes within the female infertility network in the maintenance of female fertility.**

| **Region** | **S. No.** | **Gene (NCBI Gene ID)** | **Official Name** | **Cytogenetic location** | **Exons** | **Literature related to role in female fertility** | **References** |
| --- | --- | --- | --- | --- | --- | --- | --- |
| 6q2 | 1 | *AFDN* (4301) | Afadin, adherens junction formation factor | 6q27 | 35 | *AFDN* expression was upregulated in oocytes which were matured in-vitro | **[81]** |
|  | 2 | *ESR1* (2099) | Estrogen receptor 1 | 6q25.1-q25.2 | 22 | *ESR1* genetic variants were associated with fertility status in females with endometriosis | **[82]** |
|  | 3 | *EZR* (7430) | Ezrin | 6q25.3 | 14 | ERM proteins necessary for blastocyst implantation | **[83]** |
|  | 4 | *FBXO5* (26271) | F-box protein 5 | 6q25.2 | 6 | *FBXO5* expression was downregulated in older oocytes (from females above 40 years age) compared to younger oocytes (from females under 32 years of age) | **[84]** |
|  | 5 | *GTF2H5* (404672) | General transcription factor IIH subunit 5 | 6q25.3 | 3 | High expression of *GTF2H5* was observed in human MII oocytes and blastocyst | **[85]** |
|  | 6 | *NUP43* (348995) | Nucleoporin 43 | 6q25.1 | 11 | *NUP43* was a component of functional gene cluster which was highly represented in engineered human ectocervical tissue treated with luteal phase hormones | **[86]** |
|  | 7 | *PRKN* (5071) | Parkin RBR E3 ubiquitin protein ligase | 6q26 | 13 | *PRKN* expression was reduced in aged oocytes (from 11 months old mice) compared with young oocytes (from mice aged 8 to 12 weeks) | **[87]** |
|  | 8 | ***PSMB1***(5689) | Proteasome 20S subunit beta 1 | 6q27 | 6 | A copy number variation identified in the 6q27 region (containing *PSMB1*) in a patient with premature ovarian failure | **[22]** |
|  | 9 | *RAET1E* (135250)* | Retinoic acid early transcript 1E | 6q25.1 | 10 | *Raet1e* expression was downregulated in rat granulosa cells treated with human hFSH compared with granulosa cells which were not given hFSH treatment | **[88]** |
|  | 10 | *RAET1L* (154064) | Retinoic acid early transcript 1L | 6q25.1 | 5 | *RAET1L* was a component of functional gene cluster which was highly represented in engineered human ectocervical tissue treated with follicular phase hormones | **[86]** |
|  | 11 | *RGS17* (26575) | Regulator of G protein signaling 17 | 6q25.2 | 7 | *RGS17* was more enriched in the granulosa cell transcriptome compared with theca cell transcriptome | **[89]** |
|  | 12 | *RPS6KA2* (6196) | Ribosomal protein S6 kinase A2 | 6q27 | 29 | *RPS6KA2* mRNA was detected in cumulus cells of females undergoing COS with ICSI | **[90]** |
|  | 13 | *SNX9* (51429)* | Sorting nexin 9 | 6q25.3 | 19 | *Snx9* was downregulated in blastocysts produced by IVF compared with blastocysts fertilized *in vivo* | **[91]** |
|  | 14 | *SYNE1* (23345) | Spectrin repeat containing nuclear envelope protein 1 | 6q25.2 | 153 | A *SYNE1* genetic variant was found to be significantly associated with endometriosis risk in a meta-analysis of 11 GWAS studies | **[92]** |
|  | 15 | *TAB2* (23118) | TGF-beta activated kinase 1 (MAP3K7) binding protein 2 | 6q25.1 | 15 | Increased expression of *TAB2* in granulosa cells of women with PCOS without evidence for insulin resistance (PCOS-IR) | **[93]** |
|  | 16 | *ULBP1* (80329) | UL16 binding protein 1 | 6q25.1 | 5 | The expression of *ULBP1* was upregulated in cumulus granulosa cells compared to floating granulosa cells | **[94]** |
|  | 17 | *ULBP2* (80328) | UL16 binding protein 2 | 6q25.1 | 5 | The levels of *ULBP2* in the peritoneal fluid were significantly increased in females with deep infiltrating endometriosis compared with healthy controls | **[95]** |
|  | 18 | *ULBP3* (79465) | UL16 binding protein 3 | 6q25.1 | 5 | *ULBP3* expression was decreased in ectopic endometrial cells compared to eutopic endometrial cells with/without endometriosis | **[96]** |
| 16q2 | 19 | *BCAR1* (9564) | Breast cancer antiestrogen resistance 1 | 16q23.1 | 14 | Differential binding of *BCAR1* with PGR was observed in endometrial biopsies from fertile women taken during the proliferative and mid-secretory phases | **[97]** |
|  | 20 | *CENPN* (55839) | Centromere protein N | 16q23.2 | 13 | *CENPN* is located near a single nucleotide polymorphism which was found to be significantly associated with embryonic aneuploidy in Caucasian females undergoing IVF treatment | **[98]** |
|  | 21 | *COX4I1* (1327) | Cytochrome c oxidase subunit 4I1 | 16q24.1 | 6 | *COX4I* expression was upregulated in older oocytes (from females above 40 years age) compared to younger oocytes (from females under 32 years of age) | **[84]** |
|  | 22 | *FBXO31* (79791) | F-box protein 31 | 16q24.2 | 11 | *FBXO31* was predicted to be a target gene for microRNAs differentially expressed between human cumulus granulosa cells from females with PCOS compared with normal females | **[99]** |
|  | 23 | ***IRF8***(3394) | Interferon regulatory factor 8 | 16q24.1 | 10 | *IRF8* and *MEF2C* are regulated at both transcriptional and translational levels in the endometrial epithelium during the window of implantation | **[24]** |
|  |  |  |  |  |  | Increase in *IRF8*-positive cells during the proliferative phase of menstrual cycle in women with endometriosis | **[23]** |
|  | 24 | *MAP1LC3B* (81631) | Microtubule associated protein 1 light chain 3 beta | 16q24.2 | 4 | The expression of MAP1LC3B was increased in FGSCs exposed to spermidine | **[100]** |
|  | 25 | *OSGIN1* (29948)* | Oxidative stress induced growth inhibitor 1 | 16q23.3 | 6 | *OSGIN1* expression was significantly downregulated in a rat model of PCOS treated with 5α-dihydrotestosterone compared with control mice | **[101]** |
|  | 26 | *PLCG2* (5336) | Phospholipase C gamma 2 | 16q23.3 | 33 | *PLCG2* expression was upregulated in older oocytes (from females above 40 years age) compared to younger oocytes (from females under 32 years of age) | **[84]** |
|  | 27 | *PSMD7* (5713) | Proteasome 26S subunit, non-ATPase 7 | 16q23.1 | 7 | The expression of *PSMD7* was significantly different between females with endometriosis and healthy controls | **[102]** |
|  | 28 | *USP10* (9100) | Ubiquitin specific peptidase 10 | 16q24.1 | 19 | USP10 promotes proliferation and migration of endometrial stromal cells in endometriosis while inhibiting their apoptosis | **[103]** |
|  | 29 | *ZNRF1* (84937) | Zinc and ring finger 1 | 16q23.1 | 7 | *ZNRF1* was predicted to be a target gene for has-let-7b-5p, a small non-coding RNA which was downregulated in 8-cell stage compared with blastocyst stage | **[104]** |
| Xq2 | 30 | *AIFM1* (9131)* | Apoptosis inducing factor mitochondria associated 1 | Xq26.1 | 18 | *LONP1* controls oocyte development by interacting with *AIFM1* and preventing its translocation from the cytoplasm to the nucleus in mice | **[105]** |
|  | 31 | *AMOT* (154796)* | Angiomotin | Xq23 | 17 | *Amot* was reported to be play a role in the proliferation and differentiation of mice uterine cells during the post-implantation period | **[106]** |
|  | 32 | *ATG4A* (115201) | Autophagy related 4A cysteine peptidase | Xq22.3 | 15 | *ATG4A* expression was reported to be highest in primordial follicle stage with subsequent reduction in later stages | **[107]** |
|  | 33 | *BCAP31* (10134) | B cell receptor associated protein 31 | Xq28 | 9 | The expression of *BCAP31* was more frequently observed in euploid embryos which failed to implant compared to the embryos that implanted successfully | **[108]** |
|  | 34 | *CD40LG* (959)* | CD40 ligand | Xq26.3 | 5 | In utero exposure of BPA led to a decrease in the expression of *Cd40lg* post-natal day 4 mice ovaries. BPA exposure resulted in reduced fertility in the female mice | **[109]** |
|  | 35 | *CENPI* (2491) | Centromere protein I | Xq22.1 | 26 | *CENPI* is a candidate gene for primary ovarian insufficiency | **[110]** |
|  | 36 | *CETN2* (1069) | Centrin 2 | Xq28 | 5 | A deletion in Xq28 region including the *CETN2* gene was identified in 2 females with POI | **[111]** |
|  | 37 | *COL4A5* (1287) | Collagen type IV alpha 5 chain | Xq22.3 | 57 | Deletions within the *COL4A5-COL4A6* genes were identified in patients with uterine leiomyomas | **[112]** |
|  | 38 | *COL4A6* (1288) | Collagen type IV alpha 6 chain | Xq22.3 | 50 | Deletions within the *COL4A5-COL4A6* genes were identified in patients with uterine leiomyomas | **[112]** |
|  | 39 | *CSTF2* (1478) | Cleavage stimulation factor subunit 2 | Xq22.1 | 16 | *CSTF2* was among several genes which were significantly downregulated in slow-frozen MII oocytes compared with fresh oocytes | **[113]** |
|  | 40 | *CUL4B* (8450)* | Cullin 4B | Xq24 | 24 | Knockout of *Cul4b* results in embryonic lethality and abnormal placental development in mice | **[114]** |
|  | 41 | *ELF4* (2000) | E74 like ETS transcription factor 4 | Xq26.1 | 12 | *ELF4* expression was significantly downregulated in oocytes from older females (above 40 years age) compared with oocytes from younger females (below 30 years) | **[115]** |
|  | 42 | *FLNA* (2316)* | Filamin A | Xq28 | 48 | FLNA may play a role in remodeling the actin cytoskeleton in UECs and ESCs required for implantation in rat uterus | **[116]** |
|  | 43 | *FMR1* (2332) | FMRP translational regulator 1 | Xq27.3 | 17 | Women with premutation levels of CGG triplet repeats in *FMR1* (55 to 199 repeats) have increased risk of POF | **[117]** |
|  | 44 | *GAB3* (139716) | GRB2 associated binding protein 3 | Xq28 | 11 | *Gab3* was reported to be essential for tumor cell clearance and successful completion of pregnancy in mice | **[118]** |
|  | 45 | *GABRA3* (2556) | Gamma-aminobutyric acid type A receptor subunit alpha 3 | Xq28 | 10 | *GABRA3* was reported to be differentially expressed between the obese women with PCOS group and obese women without PCOS group | **[119]** |
|  | 46 | *GABRQ* (55879)* | Gamma-aminobutyric acid type A receptor subunit theta | Xq28 | 10 | The expression of *Gabrq* was significantly downregulated in the brain of obese anovulatory mice (insulin-resistant and leptin-deficient) 7 days after receiving fat transplant from ovulating mice | **[120]** |
|  | 47 | *HAUS7* (55559) | HAUS augmin like complex subunit 7 | Xq28 | 14 | A deletion in Xq28 region including the *HAUS7* gene was identified in 2 females with POI | **[111]** |
|  | 48 | *HCFC1* (3054) | Host cell factor C1 | Xq28 | 26 | *HCFC1* expression was decreased after 6 days of culture in blastocysts from advanced maternal age infertile females compared with fertile females | **[121]** |
|  | 49 | *HMGB3* (3149) | High mobility group box 3 | Xq28 | 8 | *HMGB3* was differentially expressed in the endometrial tissues of females with tubal factor infertility, compared to healthy females | **[122]** |
|  | 50 | *HNRNPH2* (3188) | Heterogeneous nuclear ribonucleoprotein H2 | Xq22.1 | 2 | The *HNRNPH2* was predicted to be a potential target of *ARMCX3-AS1*, a long noncoding RNA whose expression was dysregulated in ectopic endometrial tissue, compared with eutopic endometrial tissue, both taken from females with endometriosis | **[123]** |
|  | 51 | *IDH3G* (3421) | Isocitrate dehydrogenase (NAD(+)) 3 non-catalytic subunit gamma | Xq28 | 12 | A partial Xq deletion encompassing the Xq27.3-q28 region (containing *IDH3G*) was identified in a female with premature ovarian failure | **[124]** |
|  | 52 | *IKBKG* (8517) | Inhibitor of nuclear factor kappa B kinase regulatory subunit gamma | Xq28 | 13 | The levels of miR-134 were higher in follicular fluid from older women compared with younger women. *IKBKG* was predicted to be a target gene for miR-134. Higher levels of miR-134 may lead to decreased expression of *IKBKG* in older females | **[125]** |
|  | 53 | *IRAK1* (3654)* | Interleukin 1 receptor associated kinase 1 | Xq28 | 15 | *IRAK1* was significantly downregulated in germinal vesicle oocytes of older mice (aged 32 weeks) compared with young mice (aged 5 weeks) | **[126]** |
|  | 54 | *IRS4* (8471) | Insulin receptor substrate 4 | Xq22.3 | 3 | *IRS4* was reported to be overexpressed in females with uterine leiomyomas harboring COL4A5-COL4A6 deletions | **[112]** |
|  | 55 | *L1CAM* (3897) | L1 cell adhesion molecule | Xq28 | 29 | *L1CAM* expression was significantly higher in patients with endometriosis compared to healthy controls | **[127]** |
|  | 56 | *MTM1* (4534) | Myotubularin 1 | Xq28 | 19 | *MTM1* expression was significantly upregulated in the ovarian cortex of females with PCOS and testosterone-treated human ovarian granulosa-like tumor cells, compared with healthy controls | **[128]** |
|  | 57 | *MTMR1* (8776) | Myotubularin related protein 1 | Xq28 | 23 | A partial Xq deletion encompassing the Xq27.3-q28 region (containing *UBL4A*) was identified in a female with premature ovarian failure | **[124]** |
|  | 58 | *MORF4L2* (9643) | Mortality factor 4 like 2 | Xq22.2 | 6 | *MORF4L2* was predicted to be a potential target of *XLOC_008200*, a long noncoding RNA whose expression was dysregulated in ectopic endometrial tissue, compared with eutopic endometrial tissue, both taken from females with endometriosis | **[123]** |
|  | 59 | *NXF5* (55998) | Nuclear RNA export factor 5 | Xq22.1 | 20 | *NXF5*, a functional homolog of *FMR1*, affected by t(X;15)(Xq22;p11) translocation in a girl with primary amenorrhea | **[110]** |
|  | 60 | *PSMD10* (5716) | Proteasome 26S subunit, non-ATPase 10 | Xq22.3 | 5 | The expression of *PSMD10* was significantly different between females with endometriosis and healthy controls | **[102]** |
|  | 61 | *RAP2C* (57826) | RAP2C, member of RAS oncogene family | Xq26.2 | 9 | *RAP2C* variants were reported to be associated with duration of pregnancy in a genome-wide association study of 43,568 European women | **[129]** |
|  |  |  |  |  |  | *RAP2C* was identified as a hub gene in a RIF protein-protein interaction network | **[130]** |
|  | 62 | *RPL10* (6134) | Ribosomal protein L10 | Xq28 | 7 | Several mutations were identified in *RPL10* (also known as *QM*) in a patient with POF | **[131]** |
|  | 63 | *SLC25A5* (292) | Solute carrier family 25 member 5 | Xq24 | 4 | A 239-kb Xq24 microdeletion involving *SLC25A5* detected in a woman with sXCI with a history of recurrent pregnancy loss | **[132]** |
|  | 64 | *UBE2A* (7319)* | Ubiquitin conjugating enzyme E2 A | Xq24 | 6 | *Ube2a-/-* female mice are infertile and not able to produce litters | **[133]** |
|  | 65 | *UBL4A* (8266) | Ubiquitin like 4A | Xq28 | 4 | A partial Xq deletion encompassing the Xq27.3-q28 region (containing *UBL4A*) was identified in a female with premature ovarian failure | **[124]** |
|  | 66 | ***UPF3B***(65109)* | Regulator of nonsense transcripts 3B | Xq24 | 16 | *Upf3b* predicted as a target gene for rno-miR-141-5p microRNA, a noncoding RNA that may play a role in modulating endometrial receptivity in rats with endometriosis | **[25]** |
|  | 67 | *VBP1* (7411) | VHL binding protein 1 | Xq28 | 8 | A gain of an approximately 73.5 kb region, including the *VBP1* gene, was identified in a patient with uterine leiomyoma | **[134]** |
|  | 68 | *XIAP* (331) | X-linked inhibitor of apoptosis | Xq25 | 10 | *XIAP* mRNA expression was increased in the endometrium of females with tubal infertility undergoing IVF. Successful pregnancy after IVF treatment was observed in females with lower expression of *XIAP* mRNA | **[135]** |

Hub genes are highlighted in bold, * - Reports including animal models, *ARMCX3-AS1*: ARMCX3 antisense RNA 1; BPA: Bisphenol A; COS: Controlled ovarian stimulation; ESCs: Endometrial stromal cells; EZR: Ezrin/Radixin/Moesin; FGSCs: Female germline stem cells; GWAS: Genome-wide association study; hFSH: Human follicular stimulating hormone; ICSI: Intracytoplasmic sperm injection; IVF: In-vitro fertilization; *LONP1*: Lon peptidase 1, mitochondrial; *MEF2C*: Myocyte enhancer factor 2C; MII: Meiosis II; PCOS: Polycystic ovary syndrome; PCOS-IR: PCOS without evidence for insulin resistance; PGR: Progesterone receptor; POF: Premature ovarian failure; POI: Primary ovarian insufficiency; RIF: Recurrent implantation failure; rno: Rattus norvegicus; sXCI: Skewed X-chromosome inactivation; UECs: Uterine epithelial cells
